# Multivalent sulphur-modified biosilica nanostructures for bacterial enteritis therapy

**DOI:** 10.1016/j.apsb.2025.12.045

**Published:** 2026-01-01

**Authors:** Tongyi Zhao, Xiaoxi Fan, Haijia Hou, Miao Xu, Yuman Sun, Jingjie Sun, Ziwei Hao, Xuchun Chen, Long He, Xuting Zheng, Heran Li, Jiali Han

**Affiliations:** a School of Pharmacy, China Medical University, Shenyang 110122, China; b Department of Otorhinolaryngology, The First Hospital of China Medical University, Shenyang 110001, China; c Department of Thoracic Surgery, The First Hospital of China Medical University, Shenyang 110001, China; d Department of Respiratory and Critical Care Medicine, The First Hospital of China Medical University, Shenyang 110001, China; e Department of Organ Transplantation and Hepatobiliary Surgery, The First Hospital of China Medical University, Shenyang 110001, China; f Organ Transplantation Center, General Hospital of Northern Theater Command, Shenyang 110010, China; g Department of Infectious Disease, The First Hospital of China Medical University, Shenyang 110001, China

**Keywords:** Multivalent sulphur-modification, Biosilica nanostructures, Bacterial enteritis, Oral delivery, Mesoporous silica nanostructure, Inflammation, Nanomedicine, Enteric disease

## Abstract

Bacterial enteritis is a specific gastrointestinal tract disorder caused by pathogenic bacterial infection, which not only disrupts the commensal microbiota but also contributes to cascaded complications. Here, we prepared polyethyleneimine (PEI)-based mesoporous silica nanostructures, co-modified with –SH and –S–S– groups, to simultaneously eradicate the pathogenic bacteria, regulate the immune response, and reprogram the inflammatory microenvironment in the infected intestine. Referring to the multivalent sulfur modification, the –S–S– group, with its oxidizability, perturbs the glutathione balance within bacteria, while the combined reductive capacity of –SH and –S–S– scavenges excessive reactive oxygen species and mitigates inflammation-induced damage. Additionally, the well-developed nanopores with a positively charged PEI network facilitate the absorption of bacterial lipopolysaccharide, lipopeptides, flagella and cell-free DNA through hydrogen bonding and electrostatic interactions. Furthermore, the biosilica nanostructures enable the efficient encapsulation of conventional antibacterial agents, such as berberine chloride and norfloxacin, thereby achieving targeted delivery and reducing side effects, which represents a promising strategy for next-generation antimicrobial therapies.

## Graphical abstract

Intelligent MSN, functionalized with both –SH and –S–S– groups, adsorbs pathogenic factors, eliminates ROS and enables stimuli-responsive drug delivery for efficient treatment of bacterial enteritis.

**Figure:**
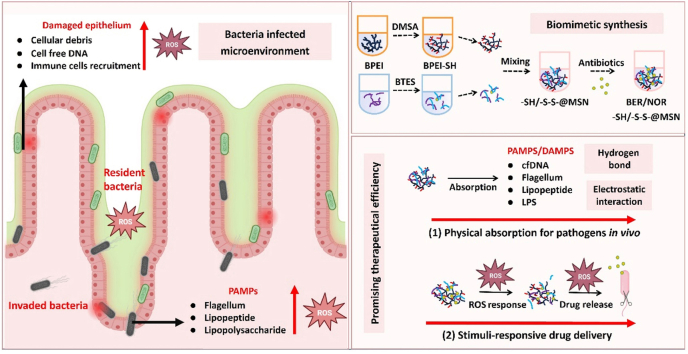

## 1 Introduction

Bacterial enteritis is a highly infectious and acute gastrointestinal disorder characterized by the abrupt onset of frequent diarrhea, fever, abdominal cramping, and hematochezia following exposure to the easily accessible pathogenic bacteria, remaining a significant epidemiological burden^1^^,^^2^. The principal causative agents—*Shigella*, *Campylobacter*, *Yersinia*, *Salmonella*, and *Escherichia coli*—exhibit invasive and pro-inflammatory phenotypes, culminating in the disruption of intestinal epithelial integrity and the presence of hemorrhagic diarrhea, along with the mucoid diarrhea^3^, ^4^, ^5^. Beyond the aforementioned clinical manifestations, severe life-threatening complications such as toxic megacolon, intestinal perforation, rectal prolapse, and pseudomembranous colitis may occur owing to the prolonged toxin accumulation and the perpetuation of an uncontrolled inflammatory microenvironment^6^^,^^7^. In worsening scenarios, bacterial toxins can translocate into the circulatory system, precipitating systemic complications such as seizures, encephalopathy, and sepsis, carrying a mortality rate even exceeds that of certain malignancies.

Upon bacterial invasion, some species, such as enter-invasive *Escherichia coli* (EIEC), can directly exploit host epithelial cells as replicative niches, hijacking intracellular signaling pathways to evade immune detection and propagate infection. Secreted exotoxin disrupts host protein synthesis, thereby promoting ulcer formation^8^, ^9^, ^10^. Conversely, enterotoxigenic *Escherichia coli* (ETEC) and *Staphylococcus aureus* colonize the mucous membrane and proliferate without directly invading or compromising epithelial integrity, but mainly induce profuse secretory diarrhea *via* electrolyte disruption^11^. Notably, lipopolysaccharide (LPS), an endotoxin derived from the outer membrane of all the Gram-negative bacteria aforementioned, is recognized as a potent activator of the innate immune response^12^. Prolonged bacterial colonization in the absence of effective clearance leads to excessive LPS accumulation, which in turn activates immune cells and exacerbates cellular injury through the toll-like receptor-4 (TLR4) binding^13^^,^^14^. Recruitment of immune cells, such as neutrophils and phagocytic macrophages, serves as a potent defense against exogenous pathogens. However, excessive levels of reactive oxygen species (ROS), including hydrogen peroxide, hydroxyl radical, and superoxide anion, generated by the respiration of immune cells, further damage cellular protein, lipids, and DNA, ultimately contributing to oxidative stress and tissue dysfunction^15^^,^^16^. In parallel, fragmented cells from the host or bacteria typically release large amounts of cell-free DNA (cfDNA), which represents a potent damage-associated molecular pattern (DAMP) or pathogen-associated molecular pattern (PAMP) to activate the inflammation cascade through toll-like receptor-9 (TLR9)^17^. In addition, specific subcellular fractions of pathogenic bacteria, such as lipopeptides and flagellum, can activate the toll-like receptor-1 or 2 (TLR1/2) and toll-like receptor-5 (TLR5) simultaneously, thus promoting a vicious inflammatory cycle^18^. Beyond the pathological features of hosts, it is worth noting that glutathione (GSH) is highly expressed within the Gram-negative bacteria, which functions in balancing oxidative stress, maintaining homeostasis, and resisting poison. The concentration of GSH in bacteria has been reported to be around 0.1–10 mmol/L and is mainly distributed l heterogeneously in the cytoso^19^^,^^20^. In brief, the resident bacteria that release toxins and the subcellular fractions, the associated infection microenvironment, characterized by elevated levels of GSH, ROS, and cfDNA, as well as the downstream inflammatory cascades, deserve to be considered as the therapeutic targets for manipulating the disease progression.

Quinolone antibiotics, such as norfloxacin (NOR), are recommended for intestinal infection therapy. Besides, antibacterial agents such as berberine chloride (BER), which is extracted from *Coptis chinensis*, show excellent antibacterial efficacy, especially against *Escherichia coli* and *Shigella*^21^. From the perspective of drug absorption, a majority of antibacterial and anti-inflammatory drugs, including BER and NOR, experience poor solubility and stability in the gastrointestinal tract (GIT), and undesirably low absorption after oral administration, accompanied by weak therapeutic efficacy and high resistance^22^^,^^23^. The systemic side effects, such as the allergic reaction and severe neurotoxicity caused by the inappropriate dosage of antibiotics, cannot be neglected either^24^^,^^25^. Moreover, these drugs cannot eradicate excessive PAMP, DAMP, and ROS simultaneously, thus showing undesirable capacity for manipulating the inflammatory microenvironment and promoting the persistence of chronic inflammation and complications. Recent years have witnessed considerable progress in the development of novel nanomedicines for the treatment of bacterial infection^24^^,^^25^. For example, Zhao et al.^26^ developed a mesoporous silica nanoparticle coated with succinylated casein to deliver a site mutation potent bactericide (T7E21R-HD5) to treat intestinal bacterial infection, which was capable of inactivating *E. coli in vivo*. Tang et al.^27^ designed a chimeric antigen receptor (CAR) mRNA-loaded nanoparticle to manipulate the macrophages *in situ*, hence facilitating the eradication of methicillin-resistant *Staphylococcus aureus* (MRSA). Most recently, Fu et al.^28^ developed an enrofloxacin and astaxanthin-loaded mesoporous hollow silica nanoparticle, which was functionalized with a cell-penetrating peptide (CPP) to facilitate the drug-targeted delivery system. This platform can reduce intestinal inflammation as well as tissue damage and improve the survival rate of *Micropterus salmoides* that suffer from enteritis. However, these systems typically involve sophisticated synthetic processes and cannot simultaneously eradicate the bacteria, attenuating the excessive immune response and reprogramming the infection microenvironment, hence leaving hidden dangers for the recurrence of the disease.

**Figure 1:**
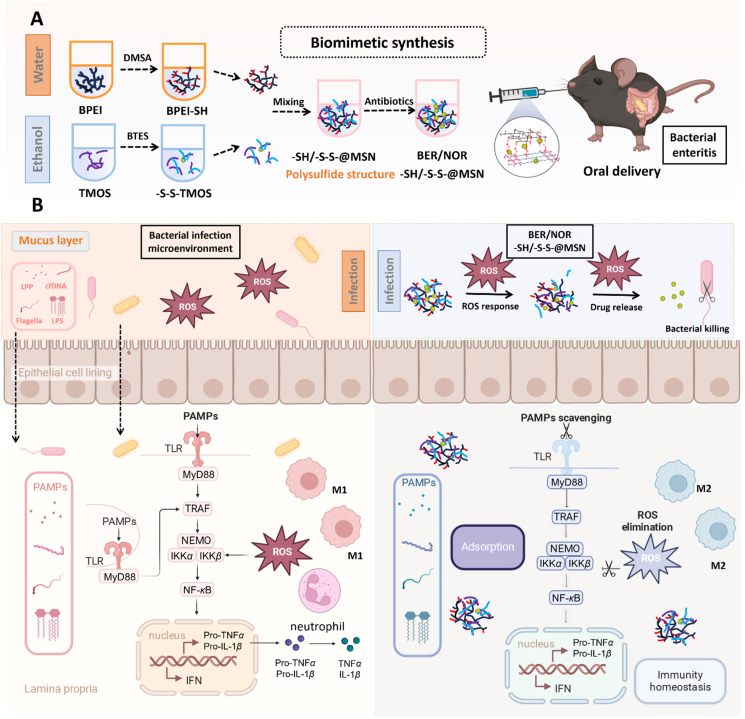
Schematic illustration of –SH/–S–S–@MSN in bacterial enteritis therapy. (A) The biomimetic synthesis route of –SH/–S–S–@MSN. (B) Therapeutic mechanism of proposed –SH/–S–S–@MSN and BER/NOR–SH/–S–S–@MSN for bacterial enteritis therapy, centering on absorption of subcellular components of bacteria (lipopeptide, LPS, flagella, and cfDNA) to reprogram the inflammatory microenvironment and inhibit the activation of pro-inflammatory signaling pathways. This picture created with BioRender. Li, H. (2025) https://BioRender.com/qg9kjtx.

In the present study, a polyethyleneimine (PEI)-based mesoporous silica nanostructure (MSN), co-modified with sulfhydryl (–SH) and disulfide (–S–S–) groups (–SH/–S–S–@MSN), was prepared through a biomimetic program at neutral pH and ambient conditions within minutes, aiming at simultaneously eradicating the pathogenic bacteria, regulating the immune response and reprograming the inflammatory microenvironment of the infected intestine (Fig. 1A). Structurally, the –S–S– with oxidizability perturbed the glutathione (GSH) balance within the bacteria, while the combined reductive capacity of –SH and –S–S– scavenged excessive ROS. Additionally, the well-developed nanopores exhibiting silanol groups along with the positively charged PEI network facilitated the absorption of various inflammatory mediators, including LPS, lipopeptide, flagella, and bacterial/host cfDNA through pore absorbing, hydrogen bonding, and electrostatic interactions. We first systematically characterized the structural and physicochemical features of MSNs with different conjugating modes, followed by investigating their capacities in eliminating multiple pathogenic factors and regulating the inflammatory microenvironment. As the nanostructures were designed to be administered orally, the mucus penetration, intestinal absorption, GIT retention profile, and bio-safety of MSNs were assessed *ex vivo* and *in vivo*. To optimize the therapeutic efficacy, anti-bacterial drugs, including NOR and BER, were loaded into the nanostructures; the loading efficiency, dispersing patterns, releasing profile, and anti-bacterial ability were meticulously evaluated. Most importantly, the therapeutic efficacy and mechanism of drug-loaded nanostructures were investigated in bacteria-infected mice, which revealed their promising therapeutic efficiency with respect to killing bacteria, alleviating inflammatory response, and reprogramming pathological microenvironment (Fig. 1B). Therefore, our work provides a practical and multipurpose therapeutic strategy for bacterial enteritis treatment with the potential for clinical adaptation.

## 2 Materials and methods

### 2.1 Ethical statement

All procedures involving animals in our research adhered strictly to institutional and national ethical guidelines. Experimental protocols were reviewed and approved by the Animal Ethics Committee of China Medical University (Protocol No. CMU20240642). Animals were housed in a specific pathogen-free (SPF) facility, maintained in accordance with the standardized protocols mandated by the Experimental Animal Ethics Committee of China Medical University.

### 2.2 Materials

Branched PEI (MW 20,000) and tetramethoxysilane (TMOS) were brought from Xiya Reagent (Chengdu, China). 2,3-Dimercaptosuccinic acid (DMSA), bis[3-(triethoxysilyl)propyl]tetrasulfide (BTES), and (3-aminopropyl)triethoxysilane (APTES) were purchased from Shanghai Rhawn Chemical Technology Co., Ltd. (Shanghai, China). Fluorescein isothiocyanate (FITC) and rhodamine isothiocyanate (RITC) were provided by Beijing Bailingwei Technology Co., Ltd. (Beijing, China). Berberine chloride (BER) and norfloxacin (NOR) were purchased from Beijing Huawei Ruike Chemical Technology Co., Ltd. (Beijing, China).

### 2.3 Preparation of BPEI–SH solution

To synthesize BPEI–SH agglomerates, 0.4 g of BPEI and 0.45 g of DMSA were dissolved in 40 mL of deionized water, followed by the addition of 300 mg NaOH to facilitate the dissolution of DMSA. The system was balanced at normal temperature and pressure for 24 h.

### 2.4 Preparation of –SH/–S–S–@MSN

1 mL of BPEI–SH solution was transferred into a 5 mL Eppendorf tube, which was introduced with 2.2 mL of inorganic-organic silica precursor containing 0.6 mL of TMOS, 0.6 mL of BTES, and 1 mL of ethanol. After standing the solution for minutes, the gelation time was recorded. The resultant hydrogel was dried and subsequently ground into powder to yield the final –SH/–S–S–@MSN containing both sulfhydryl and disulfide bonds. For comparison, –S–S–@MSNs containing only a disulfide bond, –SH@MSNs containing only sulfhydryl, and blank MSNs were synthesized. To be specific, –S–S–@MSNs was prepared using pure BPEI (10 mg/mL) as a template, with the addition of TMOS/BTES mixture, –SH@MSNs was prepared using BPEI–SH solution and TMOS, while MSN was prepared using BPEI and TMOS.

### 2.5 Characterization of –SH/–S–S–@MSN

The morphological features and particle sizes of MSNs were evaluated using TEM (Thermo Scientific, Talos F200X) and SEM (Thermo Scientific, Verios 5UC). Before imaging, 5 mg of the MSNs sample was ultrasonically dispersed in 1 mL of ethanol and deposited onto the copper grids coated with a perforated carbon film. FTIR spectra were recorded using an FTIR spectrophotometer (Spectrum 1000, Perlmer, USA) across a wavenumber range of 500–4000 cm^−1^ to identify the characteristic functional groups. Surface elemental composition, including C, N, O, Si, and S, was quantitatively assessed by an XPS instrument (ESCALAB 250Xi spectrometer, Thermo Fisher Scientific). TGA was performed using a differential thermal analyzer (HCT-1, Beijing, China) under an air atmosphere, with a temperature ramp from 25 to 700 °C. SAXD and XRD analysis were carried out on an X-ray diffractometer (EMPYREAN, PANalytical, Netherlands) using Cu–*Kα* radiation filtered with nickel at 2*θ* ranges of 0.5–10° and 5–50°, respectively. Nitrogen adsorption–desorption isotherms were obtained using a surface area analyzer (*V*_Sorb_ 2800P, APP-One, China) to assess the pore structures. The specific surface area (*S*_BET_) and average pore diameter were determined using the Brunauer–Emmett–Teller (BET) and Barrett–Joyner–Halenda (BJH) models, respectively.

### 2.6 Preparation of RITC–SH/–S–S–@MSN

For fluorescent trace, RITC-conjugated APTES (RITC-APTES) was synthesized by adding 100 μL of APTES to an ethanol solution of RITC (50 mg/mL), followed by continuous stirring in the dark for 24 h. 100 mg of each MSNs was ultrasonically dispersed in 2 mL of ethanol, then incubated with 100 μL of the RITC–APTES conjugate for another 24 h. The resulting RITC-labeled MSNs were washed repeatedly with ethanol and dried under ambient conditions.

To evaluate the fluorescence stability, the RITC-labeled MSNs were dispersed in simulated intestinal fluid (SIF) and simulated gastric fluid (SGF), respectively. After incubation for 0, 2, 6, and 12 h, the supernatants were collected by centrifugation and subjected to fluorescence analysis, with the respective media serving as baseline controls.

### 2.7 Surface/interface properties

The surface charge of MSNs was evaluated by measuring the zeta potential using a Zetasizer Lab analyzer (Malvern Instruments, UK), following complete dispersion of the nanoparticles in ethanol *via* ultrasonication. The oil–water partition coefficient (log *P*_ow_) of each MSN was determined using the classical shake-flask method. Briefly, 100 mL of *n*-octanol and 300 mL of distilled water were mixed in a thermostatic shaker at 30 °C and 100 rpm for 24 h to allow mutual saturation. The two immiscible phases were then allowed for phase separation in a separatory funnel. Next, 1 mL of RITC-labeled MSN suspension (*n* = 3) was added to the octanol-saturated aqueous phase, followed by the addition of 4 mL water-saturated *n*-octanol. The mixture was vortexed for 3 min and gently shaken at 30 °C and 100 rpm for 1 h. Fluorescence intensities in both the aqueous and organic phases were measured separately at *λ*_ex_ = 550 nm and *λ*_em_ = 620 nm, and the partition coefficient was calculated using the following Eq. (1):

(1) $$\mathrm{lg}\;P_{\text{ow}}=\mathrm{lg}\frac{F_{0}}{F_{w}}$$

where *F*_0_ and *F*_w_ represent the equilibrium fluorescence intensities of the nanostructures in *n*-octanol and water, respectively.

### 2.8 *In vitro* assay of cfDNA- and LPS scavenging capacity

To investigate the adsorptive capacity of *–*SH/*–*S*–*S*–*@MSN, RhB and EB were employed as representative molecules. A total of 30 mg of –SH/–S–S–@MSN was added to 0.02 mg/mL aqueous solutions of RhB and EB under constant stirring. At designated time intervals (0, 0.5, 1, 2, 4, and 8 h), 300 μL aliquots of the supernatant were collected. The absorbance at 554 and 611 nm was subsequently recorded using a microplate reader to quantify residual dye concentrations. The DNA adsorption ability of the MSNs was assessed using the Quant-iT™ PicoGreen™ dsDNA assay kit (Fisher Scientific). Specifically, 20 mg of –SH/–S–S–@MSN was introduced into 10 mL ctDNA/cfDNA solution (5 mg/mL) with magnetic stirring. At time points of 0, 1, 2, 3, and 4 h, 100 μL samples of the supernatant were withdrawn and incubated with an equal volume of PicoGreen working reagent. Fluorescence intensity was measured at 485 nm excitation and 520 nm emission, and DNA concentrations were determined *via* a standard calibration curve. The capacity of –SH/–S–S–@MSN to sequester LPS was quantified using a commercial LPS ELISA kit (Andy Gene, Beijing, China). For each assay, 40 mg of –SH/–S–S–@MSN was dispersed into 5 mL of LPS solution (240 ng/L, Solarbio, Beijing) under continuous stirring. At 0, 1, 2, and 3 h, 200 μL of the mixture was centrifuged, and the supernatant was analyzed according to the manufacturer's ELISA protocol. Absorbance at 450 nm was measured to determine LPS concentration based on a standard curve.

### 2.9 *In vitro* antioxidant assay

The radical scavenging activity of the MSNs was assessed using the 2,2-diphenyl-1-picrylhydrazyl (DPPH) assay (Sigma–Aldrich, USA). Ethanol-based suspensions of the MSNs at various concentrations (0, 0.625, 1.25, 2.5, and 5 mg/mL; *n* = 3) were mixed in a 1:1 ratio with 2 mL of 100 μg/mL DPPH· solution and incubated in the dark for 4 h. The absorbance of the supernatant was then measured at 520 nm. In a complementary assay, residual H_2_O_2_ was quantified *via* a horseradish peroxidase (HRP)-mediated azino-bis(3-ethylbenzothiazoline-6-sulfonic acid (ABTS) chromogenic reaction. Briefly, 500 μL of 0.1 mmol/L H_2_O_2_ was incubated with 500 μL of MSNs dispersed in PBS (pH 7.4) at various concentrations (0, 1.25, 2.5, 5, 10, and 15 mg/mL; *n* = 3) at 37 °C for 24 h. Following the addition of 100 μL of 0.1 mg/mL HRP and 100 μL of 1 mg/mL ABTS, a characteristic blue-green color developed. The resulting absorbance was recorded at 415 nm.

The superoxide radical (·$O_{2}^{-}$) scavenging ability was determined using the Superoxide Anionic Colorimetric Assay Kit (Elabscience, Wuhan). The method is based on the reduction of water-soluble tetrazolium (WST-1) by xanthine/xanthine oxidase-derived superoxide, forming a soluble formazan dye that is suppressed by antioxidant activity. For each well, 20 μL of MSNs in PBS at varying concentrations, 20 μL of enzyme reagent, and 200 μL of substrate mixture were added. After 20 min of incubation at 37 °C, the absorbance at 450 nm was measured.

Hydroxyl radical (·OH) scavenging was assessed using the Hydroxyl Free Radical Scavenging Capacity Assay Kit (Elabscience, Wuhan). Hydroxyl radicals were generated through Fenton chemistry (H_2_O_2_/Fe^2+^), and salicylic acid served as the trapping agent, forming the chromophore 2,3-dihydroxybenzoic acid. The reaction mixture comprised 100 μL each of substrate A and B, 480 μL distilled water, 200 μL substrate C, and 20 μL MSN suspension at different concentrations (*n* = 3). After 20 min of incubation at 37 °C, the absorbance at 510 nm was recorded. Finally, H_2_O_2_ scavenging efficiency was evaluated using a hydrogen peroxide colorimetric kit (Elabscience, Wuhan, China), based on the formation of a stable yellow complex with ammonium molybdate. Equal volumes (500 μL) of 0.1 mmol/L H_2_O_2_ and MSN suspensions at different concentrations (*n* = 3) were co-incubated for 24 h at 37 °C. Following the manufacturer's protocol, the absorbance was measured at 405 nm after mixing and standing. H_2_O_2_ concentrations were calculated using a standard curve.

### 2.10 Intestinal mucus penetration

To mimic the viscoelastic properties of intestinal mucus, model media consisting of cross-linked 0.5% (*w*/*v*) hydroxyethyl cellulose (HEC), viscous 80% (*v*/*v*) glycerol, and freshly collected rat intestinal mucus were prepared. Each medium (1.5 mL) was loaded into a vertically positioned syringe barrel, and 0.5 mL of SIF containing RITC-labeled MSNs (1 mg/mL, *n* = 3) was carefully layered on top. After a 12 h diffusion period at 37 °C, 0.2 mL of the bottom fraction was carefully collected. After rinsing to remove the residual matrix, fluorescence intensity was measured (*λ*_ex_ = 550 nm, *λ*_em_ = 620 nm). The penetration efficiency of the nanoparticles was calculated using the following Eq. (2):

(2) $$\text{Particle}\;\text{penetration}\;\text{ratio}\;\left(\%\right)=\frac{I_{i}}{\sum_{i=1}^{5}I_{i}}\times 100\%$$

where *I*_*i*_ represents the fluorescence intensity of the nanostructures that reached the bottom (0.2 mL segment of the medium column).

### 2.11 Multiple particle tracking

The multiple particle tracking (MPT) approach was employed to quantify the Brownian motion behavior of MSNs formulations within native intestinal mucus. Freshly collected mucus (200 μL) was gently harvested from rat intestinal lumens and incubated with 10 μL of MSNs suspension (200 μg/mL, *n* = 3) at 37 °C for 30 min to allow particle–mucin interactions. Subsequently, 20 μL of the mixture was placed on a microscopy slide, and particle trajectories were recorded in real time using CLSM at a 37 frames per second. A total acquisition time of 10 min was used for each sample. The MSD and effective diffusion coefficient (*D*_eff_) of the particles were computed based on the following Eqs. (3) and Eq. (4):

(3) $${\text{MSD}}_{\tau }={\left(x_{\left(t+\tau \right)}-x_{t}\right)}^{2}+{\left(y_{\left(t+1\right)}-y_{t}\right)}^{2}$$

(4) $$D_{\text{eff}}=\frac{MSD}{4_{\tau }}$$

where ${\text{MSD}}_{\tau }$ represents the average displacement within the time interval *τ*, *x*(*t*) and *y*(*t*) denote the particle coordinates at time *t*, *x*_*t*+1_ and *y*_*t*+1_ represent the coordinates of the particle at the next time point *t*+1, and *D*_eff_ represents the effective diffusion coefficient, which describes the rate at which particles diffuse within the medium. A higher value indicates a faster diffusion process.

### 2.12 Intestinal mucosal adhesion assay

Small intestinal segments (1 cm × 4 cm) were excised from SD rats following overnight fasting, gently rinsed with SIF, and mounted onto glass slides. RITC-labeled –SH/–S–S–@MSNs (10 mg, *n* = 3) were evenly dispersed across the mucosal surface. The samples were secured on a 45° inclined platform and incubated at 37 °C for 15 min to facilitate particle adhesion. Following incubation, the tissues were gently rinsed with SIF for 5 min to remove non-adherent nanoparticles. The wash solutions were collected, and fluorescence intensity (*λ*_ex_ = 570 nm, *λ*_em_ = 595 nm) was quantified to estimate the number of detached particles. Concurrently, fluorescence imaging of the intestinal mucosa post-washing was performed using the Living Image System (Night OWL II LB 983, Germany), and the retained RITC fluorescence was semi-quantified.

### 2.13 Intestinal retention of the MSNs

To evaluate GIT retention of the MSNs, healthy mice were fasted overnight before oral administration of RITC-labeled –SH/–S–S–@MSN at a dose of 100 mg/kg (*n* = 3). At predetermined time points, animals were euthanized, and the entire GIT, along with major organs, were harvested for *ex vivo* fluorescence imaging. Fluorescence intensities were semi-quantitatively analyzed to assess biodistribution and retention profiles.

### 2.14 Biocompatibility of the MSNs

Hemocompatibility of the MSNs was first assessed *via* a hemolysis assay. Venous blood was collected from overnight-fasted SD rats, centrifuged, and washed to isolate red blood cells (RBCs). The RBCs were then diluted with sterile saline to yield a 2% (*v*/*v*) suspension. Aliquots of 2 mL RBC suspension were mixed with equal volumes of MSN suspensions at varying concentrations (*n* = 3). Saline and deionized water were used as negative and positive controls, respectively. After incubation at room temperature for 4 h, samples were centrifuged, and the absorbance of the supernatant was measured at 540 nm to quantify hemoglobin release.

To evaluate systemic toxicity, mice were orally administered MSNs suspensions at a double dose of 200 mg/kg once daily for 14 consecutive days (*n* = 3). On Day 14, whole blood and serum samples were collected for comprehensive hematological profiling (BC-2800vet, Mindray, China) and serum biochemical analyses (poch-100i, Sysmex, Japan). Major organs, including the heart, liver, spleen, lungs, kidneys, and GIT, were excised, weighed, and subjected to histopathological examination *via* H&E staining.

### 2.15 Drug loading and characterization

BER and NOR were selected for *in situ* loading into the –SH/–S–S–@MSN. During the synthesis of the functionalized MSNs, 40 mg of BER or NOR was pre-dissolved in water *via* pH adjustment or mild heating to facilitate homogeneous incorporation, yielding BER/NOR-loaded –SH/–S–S–@MSN composites. To elucidate the physicochemical and crystalline characteristics before and after drug encapsulation, FTIR, XRD, and DSC analyses were performed. Drug release profiles were investigated *in vitro* using a ZRS-8G dissolution apparatus (Tianjin XinTianGuang Analytical Instrument Technology Co., Ltd., China), employing the small-volume dissolution method. Briefly, samples equivalent to 5 mg of loaded drug (BER/NOR and BER/NOR–SH/–S–S–@MSN, *n* = 3) were immersed in 250 mL of SIF under constant stirring at 100 rpm. Aliquots (3 mL) were withdrawn at predetermined intervals, filtered through 0.22 μm microporous membranes, and analyzed spectrophotometrically using a microplate reader in UV mode to quantify drug concentrations against calibration curves. The withdrawn volume was immediately replenished with fresh SIF to maintain sink conditions throughout the experiment.

### 2.16 *In vitro* antibacterial assay

*Escherichia coli* (ETEC O78:K80) cultures were revived and passaged to the third generation. A bacterial suspension at 1 × 10^4^ CFU/mL was prepared using a turbidity standard in sterile saline. MSNs and BER or NOR loaded –SH/–S–S–@MSN containing 1.6 mg of bulk BER or NOR were subjected to UV irradiation for 15 min before incubation. These BER or NOR loaded –SH/–S–S–@MSN were then co-incubated with the bacterial suspension at 37 °C with agitation for 4 h. Subsequently, 100 μL aliquots of the supernatants were spread onto Luria–Bertani (LB) agar plates and incubated at 37 °C under 5% CO_2_ for 24 h to allow colony formation, enabling quantitative colony counting. For growth kinetics analysis, 150 μL supernatant was collected at 0, 2, 4, 6, 8, 10, and 12 h post-co-culture. Optical density (OD) at 600 nm was measured to construct bacterial growth curves (*n* = 3).

### 2.17 Acute bacterial enteritis disease modelling

An acute bacterial enteritis model was established using *Escherichia coli* (ETEC O78:K80). Six-week-old male C57BL/6J mice (around 30 g) were pre-treated with streptomycin (5 g/L) in drinking water for three days to disrupt the indigenous gut microbiota. From Day 4 onward, control animals received thrice daily oral gavage of 0.2 mL sterile saline (0.9% NaCl), whereas the model group was orally administered with normal saline once, followed by two daily doses of ETEC suspension (1.2 × 10^12^ CFU/mL in 0.2 mL saline) for four consecutive days. Post-infection, mice were housed individually in fresh cages lined with filter paper (no bedding). Clinical symptoms, including diarrhea (evidenced by watery stools and perianal staining) and mortality, were monitored every 2 h for 4 days. To evaluate the therapeutic efficacy, MSNs and BER/NOR-loaded MSNs were orally administered 2 h after the initial bacterial inoculation. On Day 8, mice were euthanized, and serum, peritoneal fluid, intestinal tissues, and faeces were collected for cytokine quantification, histopathological examination, and 16S rRNA gene sequencing. Small intestinal samples were fixed, paraffin-embedded, and subjected to HE staining for histological evaluation. IF staining was performed using antibodies against CD86, CD206, CD31, F4/80, Ly6, caspase-3, MyD88, and NF-*κ*B. IHC analysis targeted TLR1, TLR2, TLR4, TLR5, TLR9, TNF-*α*, and IL-1*β*. Cytokine levels, including TNF-*α*, IL-1*β*, IL-*4*, IL-10, IL-17, MCP-1, MPO, TGF-*β*, and IFN-*γ*, were quantified following the manufacturer's protocols using ELISA kits (Elabscience, Wuhan, China).

### 2.18 Statistical analysis

All experiments were conducted in triplicate, except for animal studies, which included six biological replicates. Data are presented as mean ± standard deviation (SD), as indicated in the legend of each figure. Statistical comparisons between groups were performed using two-tailed Student's *t*-tests or one-way analysis of variance (ANOVA), as appropriate. Differences with *P* values less than 0.05 were deemed statistically significant.

## 3 Results and discussion

### 3.1 Synthesis and characterization of MSNs

**Figure 2:**
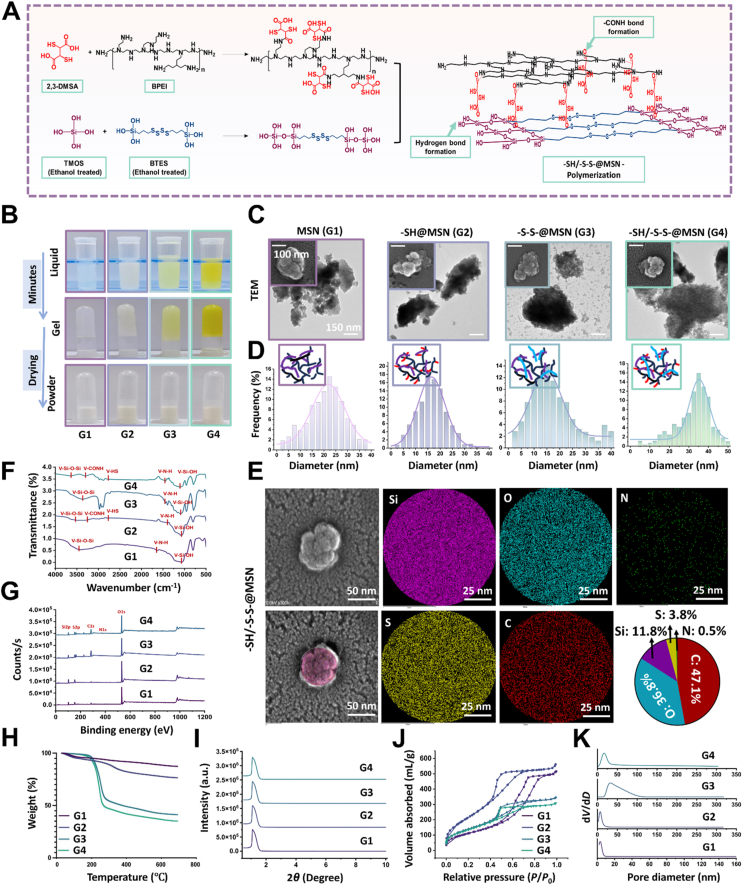
Biomimetic fabrication and characterization of –SH/–S–S–@MSN. (A) The molecular synthesis strategy of –SH/–S–S–@MSN. (B) Physicochemical appearance of MSNs in different stages. (C) TEM and SEM images of MSNs, scale bar = 100/150 nm. (D) The particle size distribution of MSNs. (E) EDS mapping images of –SH/–S–S–@MSN, scale bar = 25/50 nm (F) FTIR spectra, (G) XPS spectra, (H) TGA curves, (I) SAXD patterns, (J) Nitrogen adsorption/desorption isotherms, and (K) pore size distribution curves of MSNs.

MSNs with comparable functional moieties were biomimetic fabricated through a very simple mixing process at neutral pH and ambient conditions within minutes. The synthetic routes and different patterns of the proposed MSNs were thoroughly illustrated (Fig. 2A and B). For –SH/–S–S–@MSN, we first grafted the –SH containing 2,3-dimercaptosuccinic acid (MDSA) onto branched PEI (BPEI) through the nucleophilic substitution, accompanied by amide bond formation. Fourier transform infrared spectroscopy (FTIR) revealed N–H bending (1442 cm^−1^) and amide C=O stretching (3304 cm^−1^) bands, indicating the successful polymerization between the carboxyl group in MDSA and the amino group in BPEI. A weak absorption peak around 2732.6 cm^−1^ was assigned to the S–H stretching vibration (Fig. 2F). TMOS was applied as the silica course, which polymerizes with the disulfide-containing BTES *via* dehydration condensation. Acting as both template and catalyst, the carboxyl group in MDSA and the protonated amine group in BPEI reacted with the hydrolyzed silanol groups in the inorganic-organic TMOS–BTES complex through hydrogen bonding and electrostatic interaction, respectively, hence programming biomimetic silica deposition. Consequently, multivalent sulphur moieties were imprinted along with silica formation, bringing about the versatility of functional design. As shown in Fig. 2F, absorption peaks at 3643 cm^−1^ (*ν* O–H), 1106.8 cm^−1^ (*v* Si–O–Si), and 473 cm^−1^ (*δ*–Si–O–Si) confirmed the silica framework. Furthermore, disulfide (–S–S–) groups were endowed with an absorption band in the range of 400–600 cm^−1^. For cross-comparison, we synthesized MSN (G1), –SH@MSN (G2), –S–S–@MSN (G3) and –SH/–S–S–@MSN (G4) containing no –SH/–S–S–, only –SH, only –S–S– and –SH/–S–S–, respectively (Supporting Information Figs. S1 and S2). All synthetic processes can be controlled within several minutes under ambient conditions, where polyurethane manipulates both the reaction process and deposition space, falling into the concept of biomimetic synthesis.

**Figure 3:**
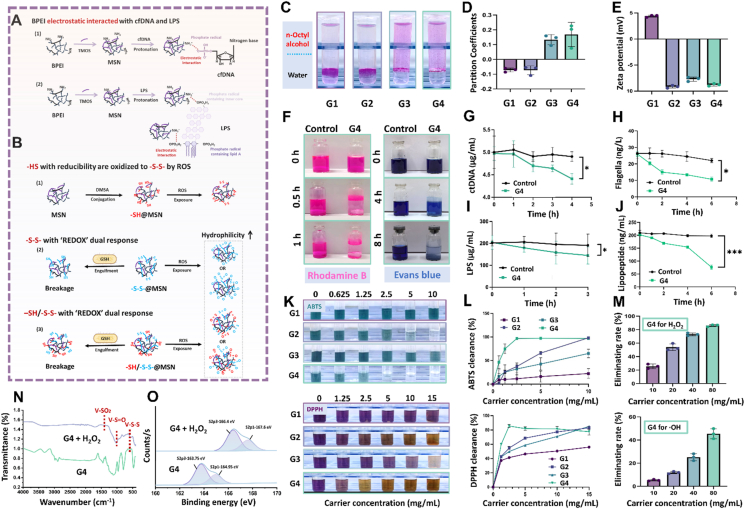
Antioxidative and *in vitro* pathogenic factors elimination capacities of –SH/–S–S–@MSN. (A) Molecular mechanism of interaction between MSNs and cfDNA/LPS. (B) ‘REDOX’ dual response property of –SH/–S–S–@MSN. (C) Oil–water partition images, (D) partition coefficients and (E) zeta potential of MSNs. (F) Time-dependent appearance of RhB and EB solutions after –SH/–S–S–@MSN co-culture. (G–J) Time-dependent cfDNA, LPS, lipopeptide, and flagellum absorption profile of –SH/–S–S–@MSN. Concentration-dependent antioxidative capacity of –SH/–S–S–@MSN, which is reflected by the color (K) and absorbance (L) change of DPPH and ABTS solutions. (M) Concentration-dependent H_2_O_2_ and ·OH elimination profiles of –SH/–S–S–@MSN. (N) FTIR and (O) XPS spectra of –SH/–S–S–@MSN before and after incubation with H_2_O_2_. Data are presented as mean ± SD (*n* = 3), ∗*P* < 0.05, and ∗∗∗*P* < 0.001. A Created in BioRender. Li, H. (2025) https://BioRender.com/qg9kjtx.

Transmission electron microscopy (TEM) and scanning electron microscopy (SEM) exhibited aggregated morphologies and porous structure of MSNs with similar average particle sizes of 20‒30 nm (Fig. 2C and D). The nitrogen adsorption/desorption isotherms of MSNs all showed typical hysteresis return lines along with the small-angle X-ray diffraction (SAXD) spectrum exhibiting well-resolved peaks at small scattering angles, indicating the presence of homogeneous mesopores (Fig. 2I–K). X-ray photoelectron spectroscopy (XPS) analysis disclosed a clear elemental peak of individual elements within all MSNs, where C, O, Si, S, and N signals were detected in –SH@MSN, –S–S–@MSN, and –SH/–S–S–@MSN (Fig. 2G, Supporting Information Fig. S3). Energy dispersive spectroscopy (EDS) mapping confirmed uniform distribution of sulfur and nitrogen across the –SH/–S–S–@MSN surface (Fig. 2E, Supporting Information Fig. S4). Moreover, the –SH/–S–S–@MSN showed the largest weight loss between 20 and 700 °C, attributed to the abundant organic functional groups, followed by –S–S–@MSN, –SH@MSN, and MSN (Fig. 2H). In addition, the obvious increase of oil-water partition from −0.072 (MSN) and −0.073 (–SH@MSN) to +0.134 (–SH/–S–S–@MSN) and +0.169 (–S–S–@MSN) indicated an enhancement of the surface lipophilicity after –S–S– polymerization (Fig. 3A–D, Supporting Information Fig. S5). After –SH and –S–S– modification, MSNs underwent decreases on surface potential charge from +4.39 to −9.14, −7.67, and −8.77 mV for –SH@MSN, –S–S–@MSN and –SH/–S–S–@MSN respectively, owning to deprotonation of hydroxyl groups derived from the MDSA (Fig. 3E).

### 3.2 REDOX response and pathogen clearance of MSNs

As far as we know, the PEI template bearing positive charges electrostatically interacted with the negatively charged PO4^3−^ groups in LPS and cfDNA. The abundant silanol groups and mesopores within the MSNs can interact and absorb the subcellular lipopeptide and flagellaof bacteria correspondingly. Furthermore, the –SH and the –S–S– moieties on MSNs with reducibility can attenuate the severe oxidative stress in the inflammatory microenvironment, while the –S–S– group with dual REDOX response can react with the abundant GSH within infected bacteria (Fig. 3A and B). First, rhodamine B (RhB) and Evans blue (EB), which possess different molecular weights and charges, were selected to study the absorption profile of –SH/–S–S–@MSN. As the molecular weight increased from around 479 Da (RhB) to 1 kDa (EB), the absorption period was correspondingly prolonged (Fig. 3F, Supporting Information Fig. S6). We then quantitatively evaluated the scavenging capacity of –SH/–S–S–@MSN against LPS, calf thymus DNA (ctDNA, to resemble the structure of cfDNA), lipopeptide, and flagella *in vitro*. As aforementioned, it is hypothesized that the protonated amine group derived from PEI electrostatically conjugates with the negative –PO_4_^3−^ groups in the DNA molecule. In parallel, the electrostatic interaction between positively charged PEI and negative –PO_4_^3−^, as well as the hydrogen bond formed between the –OH in the lipid A part and the silanol groups in MSNs, facilitated the LPS absorption. Due to the inherent mesoporous structure and enriched –OH structure of MSN, which exhibited great affinity towards the –NH_2_, –COOH, and –OH within the peptide, we considered that the lipopeptide and flagellum can either be absorbed to a large extent. As a proof of concept, we incubated the –SH/–S–S–@MSN along with the four targeted proinflammatory factors with magnetic stirring. It can be noticed that the targeted mediators were eliminated to a large extent after 4‒6 h co-incubation. Statistically, the clearance levels of MSNs against several substances (LPS, lipopeptide, flagellum, and ctDNA) were 25.12%, 70.08%, 56.2%, and 14.6%, respectively (Fig. 3G–J).

5,5′-Dithio-bis-(2-nitrobenzoic acid) (DTNB), also known as Ellman agent, was used to detect free sulfydryl (–SH) group. It reacts with R–SH, followed by forming the yellow and detectable TNB and R–S–TNB, which shows strong absorption at 412 nm^29^. As shown in Supporting Information Fig. S7, both –SH@MSN and –SH/–S–S–@MSN reacted with DTNB and generated a yellow solution, illustrating the attachment of –SH groups. After exposure to H_2_O_2_ with a gradient concentration, –SH was gradually oxidized to –S–S–, which diminished the color, which recovered through the addition of GSH with reducibility. The reversible reaction further demonstrated the successful conjugation of –SH as well as the oxidation responsiveness of –SH@MSN and –SH/–S–S–@MSN. Through self-assembly, MSNs with diversified responsive moieties were integrated to defend the inflammatory microenvironment after bacterial infection. Verifiably, ABTS and DPPH tests were carried out to qualitatively examine the oxidation resistance of MSNs. Once exposed to an oxidizing agent, colorless ABTS transforms into turquoise ABTS·^+^ free radical, where the reaction can be reversed by the antioxidant addition^30^. Similarly, DPPH neutral radical generally exhibits a dark violet color, while compounds with oxidative stability facilitate the yellow DPPH–H forming^31^. As expected, among the MSNs, –SH/–S–S–@MSN experienced the most pronounced color changes from turquoise to colorless and dark violet to yellow in ABTS and DPPH assay, respectively (Fig. 3K and L). We next incubated the –HS/–S–S–@MSN in an oxidative environment (H_2_O_2_ solution). FTIR analysis disclosed the disappearance of –SH peak around 2732.6 cm^−1^, along with the presence of sulfoxide (*ν* S=O, 1029.8 cm^−1^) and sulphone (*ν* O=S=O, 1419.4 cm^−1^) bands, confirming the responsibility of multivalent sulphur MSNs (Fig. 3N). The elevation of the binding energy in the sulfur XPS spectrum further indicated the sulfoxide and sulphone formation with +4/+6 sulfur valence (Fig. 3O, Supporting Information Fig. S8). As expected, due to the hydrophilic group formed in an oxidation environment, the degradability of –SH/–S–S–@MSNs was slightly increased (Supporting Information Fig. S9). As aforementioned, –SH with desirable reducibility directly eliminated the free radical, followed by –S–S– forming. The produced –S–S– along with the previously conjugated –S–S– bonds can be further oxidized to sulfoxide or sulphone, hence promoting the free radical elimination. Next, the capacity of –SH/–S–S–@MSN in scavenging ROS (H_2_O_2_, ·OH, and O^2−^) was quantitatively analyzed. Results revealed that –SH/–S–S–@MSN showed great sensibility towards all the free radicals in a concentration-dependent manner (Fig. 3M, Supporting Information Fig. S10).

Strikingly, versatile –S–S– also exhibited oxidability, which skillfully interrupts the GSH balance within the pathogenic bacteria's cytosol after targeted engulfment of the MSNs through electrostatic interaction. It meant that the biosilica nanostructures can simultaneously eliminate the etiologic initiators, reprogram the inflammatory microenvironment. And relieve the downstream inflammation cascade even without therapeutic drugs, thus controlling the disease progression comprehensively. For bacterial enteritis, the accumulation of pathogenic bacteria that share with higher level of GSH directly determines the profile of the downstream inflammation cascade^32^. We hypothesized that the –S–S– group in –S–S–@MSN and –SH/–S–S–@MSN can destroy the oxidative balance, homeostasis maintenance, and detoxification of bacteria through GSH elimination. The elevated degradation profile of MSNs after GSH exposure further confirmed the functional versatility of –S–S– (Fig. S9). The overall REDOX responsibility of –SH/–S–S–@MSN against the excessive ROS and GSH within the bacteria was further confirmed by the increase of hydrophilicity after exposing H_2_O_2_ and GSH-containing SIF (Supporting Information Fig. S11). To confirm the GSH eliminating capacity of the –S–S– conjugating MSN in bacteria, the supernatant of the MSN-bacteria co-culture matrix was collected for GSH quantitative analysis based on the DTNB colorimetric assay. After 4h incubation, statistically, the intracellular GSH level significantly reduced (33.78%) after functional MSN treatment, which strongly supports the reactivity of the attached –S–S– group (Supporting Information Fig. S12). Subsequently, we comprehensively analyzed the *in vitro* antibacterial capacity of MSNs, where the number of bacteria incubated was initially controlled within 1 × 10^3^ CFU per medium. After 24 h co-culture with MSNs suspension, MSNs lacking sulphur moieties exhibited the weakest antibacterial capacity. Compared to –S–S–@MSN, which primarily acts in the periplasm, –SH@MSN presented a superior eliminating profile because –SH can directly interact with the extensively distributed reductive mercaptan groups within the bacterial membrane, thus disrupting the structural integrity. Interestingly, multivalent sulphur-modified –SH/–S–S–@MSN displayed the highest antibacterial efficiency (Supporting Information Fig. S13).

### 3.3 Diffusion and permeation of MSNs in the intestinal mucus

**Figure 4:**
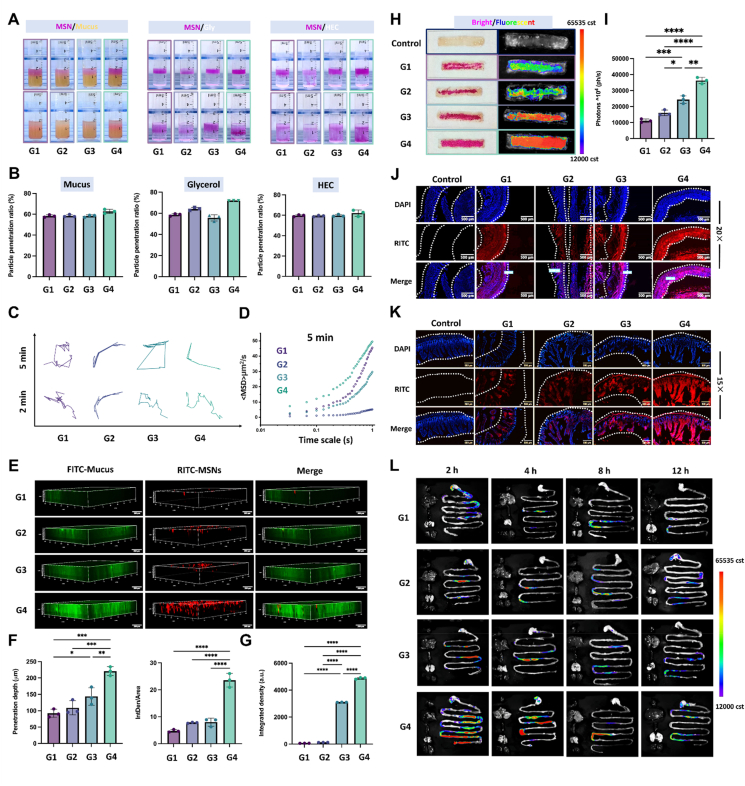
Retention and permeation of –SH/–S–S–@MSN on intestinal mucosa. (A) Diffusion profile and (B) efficiency of RITC-labeled –SH/–S–S–@MSN in fresh rat mucus, Gly, and HEC. (C) Particle trajectories and (D) MSD of MSNs within 2 and 5 min. (E) 3D CLSM images, (F) fluorescence intensity, penetration depth, and (G) 2D fluorescence intensity retained on intestinal mucus of –SH/–S–S–@MSN, scale bar = 200 μm. (H) Mucosal adhesion images and (I) fluorescence intensity of –SH/–S–S–@MSN after elution. (J) CLSM images of RITC- labeled –SH/–S–S–@MSN permeated in the *ex vivo* rat intestinal tissue. Blue: nuclei stained with DAPI, red: RITC-MSNs, scale bar = 500 μm. (K) CLSM images of –SH/–S–S–@MSN absorbed in the small intestine of mice at 2 h post oral administration. Blue: nuclei stained with DAPI, red: RITC-MSNs, scale bar = 500 μm. (L) Fluorescence images of –SH/–S–S–@MSN remained in the mice GIT at 2, 4, 8, and 12 h post oral administration. Data are presented as mean ± SD (*n* = 3), ∗*P* < 0.05, ∗∗*P* < 0.01, ∗∗∗*P* < 0.001, and ∗∗∗∗*P* < 0.0001 *vs*. indicated.

Healthy intestine comprises four-layered structures concerning the mucosa, submucosa, muscularis, and serosa^33^. Mucus secreted by the goblet cells serves as the first barrier to oral delivery, owing to the fact that the viscoelastic gel network restricts nanoparticles movement *via* adhesion and steric hindrance^34^. We first collected fresh rat mucus and investigated the mucus penetration of rhodamine isothiocyanate (RITC)-labeled MSNs placed on the top of the mucus (Fig. 4A). Following 24 h of free diffusion, –SH/–S–S–@MSN showed the best penetration profile. For MSN, the positively charged surface electrostatic attraction with the negatively charged mucus thus restricts their free movement. –SH@MSN exhibited relatively stronger mucus penetration than –S–S–@MSN. Evidently, –SH@MSN and –SH/–S–S–@MSN with reduced hydrophilicity decreased non-specific interactions with mucin proteins. Meanwhile, the reductive –SH@MSN and –SH/–S–S–@MSN cleave the disulfide bond in the mucus, facilitating diffusion. Compared to –SH@MSN, –SH/–S–S–@MSN was endowed with a balanced negative charge and hydrophobicity to achieve the most desirable penetration in mucus solution. With respect to the viscosity and space limitation of mucus, we further employed viscous glycerol and cross-linked HEC to elaborate the MSNs permeation (Fig. 4B). No significant difference was found in the permeation of MSNs in HEC, where the particle size and shape of MSNs were the determining factors, while a similar trend with that of mucus was observed in glycerol. In parallel, the particle trajectories revealed the most intense Brownian movement and the highest mean square displament (MSD) value of –SH/–S–S–@MSN in rat mucus, which was 1.09-, 1.67- and 9.61-times higher than MSN, –S–S–@MSN and –SH@MSN, respectively, within 5 min (Fig. 4C and D, Supporting Information Figs. S14 and S15).

Based on the desirable results, we incubated RITC labelled MSNs with the FITC-labeled mucus in *ex vivo* rat intestinal segments, where the complex system was comprehensively visualized and analyzed *via* confocal laser scanning microscopy (CLSM) (Fig. 4G, Supporting Information Fig. S16). It can be noted that –SH/–S–S–@MSNs were uniformly dispersed and exhibited the most potent red fluorescence signal in the mucus, followed by –S–S–@MSN, –SH@MSN, while MSN showed the poorest mucus coverage. CLSM *z*-axis scanning indicated penetrating depth from a 3D perspective (Fig. 4E and F). MSN, as well as –SH@MSN with strong hydrophilicity and undesirable mucus coverage, were mainly restricted to the top layer. In striking contrast, –SH/–S–S–@MSN penetrated longitudinally the deepest along the *z* axis, validating the excellent mucus-penetrating ability. The penetration depth of MSNs was quantitatively analyzed as 224 μm (–SH/–S–S–@MSN), 172.4 μm (–S–S–@MSN), 108.8 μm (–SH@MSN), and 92.1 μm (MSN), respectively.

### 3.4 Retention and permeability of MSNs in intestinal mucosa

Inspired by the permeation profile of MSNs within the mucus, we further explored their retention on the mucosa. The bioadhesion capacity of MSNs was evaluated through the elution method using simulated intestinal fluid. The adhesion ratio of MSN –SH@MSN, –S–S–@MSN and –SH/–S–S–@MSN were 61.13%, 68.51%, 67.27% and 86.98% at 1 min, and 8.09%, 28.78%, 46.6%, 57.56% at 5 min, respectively (Fig. 4H and I, Supporting Information Figs. S17 and S18). It was hypothesized that the optimal balance between the hydrophobicity and conjugating of the –SH groups mediated dynamic ‘SH/–S–S–’ exchange, facilitated the adhesion and deeper movement of the MSNs. RITC-labelled MSNs were utilized subsequently to visualize the retention profile. Results revealed that the majority of –SH/–S–S–@MSN remained on the mucosa after SIF elution with the highest fluorescent signal, followed by –S–S–@MSN, –SH@MSN, and MSN. Meanwhile, –SH/–S–S–@MSN was most likely to permeate into the mucosa, with the penetration depth measured to be 269.1 μm (–SH/–S–S–@MSN), 207.7 μm (–S–S–@MSN) 136.5 μm, (–SH@MSN) and 53.4 μm (MSN), respectively (Fig. 4J, Supporting Information Fig. S19).

To define the capacity of the MSNs for targeting the lesion locus, RITC labelled MSNs were orally administered to the mice, and the targeted lesion locus, *i*.*e.*, the small intestine, was extracted at 2 h after administration for CLSM imaging. Consistent with the *ex vivo* results, MSN and –SH@MSN presented a weak fluorescence signal, which was mainly restricted to the lateral margin of the intestinal villi. In sharp contrast, –SH/–S–S–@MSN greatly spread deep into the mucosa (Fig. 4K, Supporting Information Fig. S20).

### 3.5 Bio-fate, stability, and biocompatibility of MSNs

In consideration of the superiority of –SH/–S–S–@MSN in mucosa permeation, the biodistribution of the RITC-labeled MSNs was investigated through the *ex vivo* fluorescence imaging. Results revealed that MSNs started to accumulate in the small intestine at 2 h after oral administration and exhibited a promising retention profile within 12 h, with fluorescence intensity gradually declining thereafter. Notably, –SH/–S–S–@MSN exhibited the most favorable aggregation profile (Fig. 4L, Supporting Information Fig. S21). Meanwhile, all MSNs remained primarily within the GIT, with negligible signal in other main organs, aligning with targeted-therapy principles and minimizing systemic exposure. Given the oral route, the capacity for resisting the gastric digestion of four MSNs was evaluated. After immersing in the gastric acid (pH = 1) for 2 h which corresponding to the gastric resident duration, the MSNs were allowed for sufficient cleaning and drying, followed by FTIR analysis. The intact silicon network was evidenced by the persistence of Si–OH (*v* 1110 cm^−1^) and N–H (*v* 1400 cm^−1^) bands (Supporting Information Fig. S22). Next, the degradation profiles of four blank MSNs within SIF were accessed. As reported, the degradation profiles of MSN, –SH@MSN, –S–S–@MSN, and –SH/–S–S–@MSN within 24 h were 35.5%, 40.9%, 82.5% and 62.4%, respectively. Thus, the nano-foam-like MSN aggregation can gradually disintegrate and degrade to release as small cells within the intestine under the expected timeframe without eliciting severe toxicity (Supporting Information Fig. S23).

*In vitro* and *in vivo* biocompatibility evaluations were then sequentially carried out. First, MSNs were incubated with rat blood within the concentrations ranging from 0 to 400 μg/mL. It was found that all MSNs showed an excellent hemocompatibility with hemolysis ratio lower than 2% (Supporting Information Fig. S24). In particular, –SH/–S–S–@MSN showed the most favorable hemocompatibility profile. In parallel, MSNs were orally administered to mice for 14 consecutive days. No unexpected mortality or abnormal behavior was observed in any group. H&E staining confirmed no significant histopathological abnormalities or damage after oral administration of the MSNs (Supporting Information Fig. S25). In addition, all the hematological parameters are within the reference range (Supporting Information Fig. S26). In brief, despite the promising interactions within intestinal mucosa, all MSNs exhibited excellent biosafety, which was considered a prerequisite for biomedical applications.

### 3.6 Drug loading and release profile of MSNs

**Figure 5:**
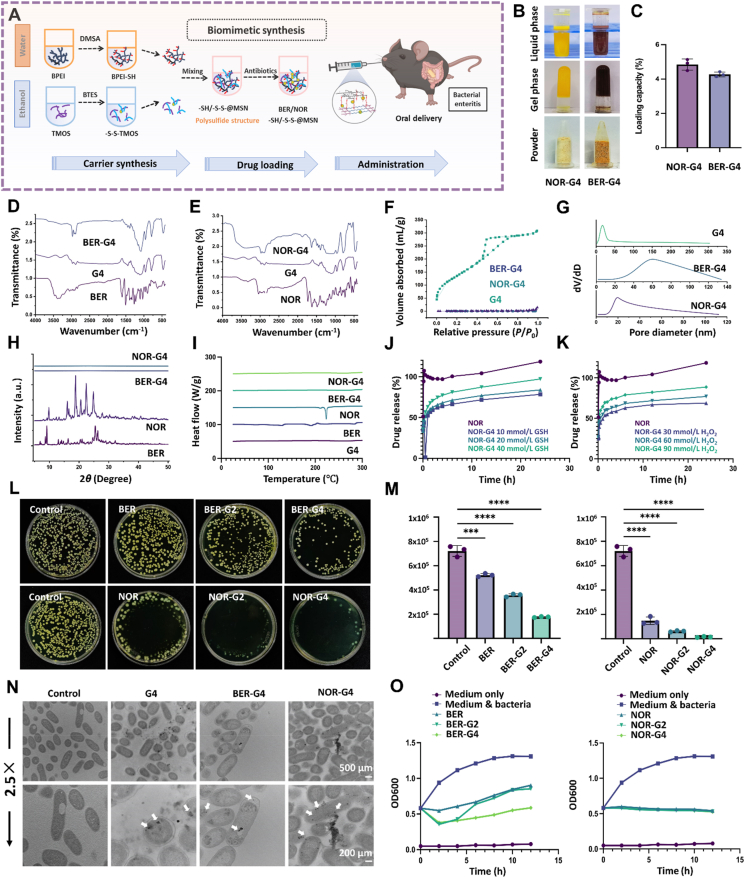
Fabrication and characterization of drug-loaded –SH/–S–S–@MSN. (A) The synthesis strategy of BER/NOR–SH/–S–S–@MSN. (B) Physiochemical appearance of drug-loaded MSNs in different stages, and (C) drug loading capacity of BER/NOR–SH/–S–S–@MSN. FTIR spectra of (D) BER–SH/–S–S–@MSN and (E) NOR–SH/–S–S–@MSN. (F) Nitrogen adsorption/desorption isotherms and (G) pore size distribution curves of drug-loaded –SH/–S–S–@MSN. (H) XRD and (I) DSC spectra of BER/NOR–SH/–S–S–@MSN. Drug release profiles of NOR–SH/–S–S–@MSN in SIF containing (J) 10, 20, and 40 mmol/L GSH or (K) 30, 60, and 90 mmol/L H_2_O_2_. (L) Images and (M) efficiency of *in vitro* antibacterial activity of BER/NOR–SH/–S–S–@MSN. (N) Bio-TEM images of *E. coli* before and after being co-cultured with –SH/–S–S–@MSN and BER/NOR–SH/–S–S–@MSN. Scale bars = 500 μm (Normal) and 200 μm (2.5 × magnification). (O) OD_600_ growth curve of normal *E. coli* before and after co-cultured with –SH/–S–S–@MSN and BER/NOR–SH/–S–S–@MSN. Data are presented as mean ± SD (*n* = 3), ∗∗∗*P* < 0.001, and ∗∗∗∗*P* < 0.0001 *vs*. indicated. A Created in BioRender. Li, H. (2025) https://BioRender.com/qg9kjtx.

The aforementioned results verified the excellent properties of SH/–S–S–@MSNs concerning intestinal adhesion, biocompatibility, and capacity in elimination of critical etiologic agents and inflammatory factors. Attributing to the porous structure, we hypothesized that –SH/–S–S–@MSN held great promise to be an efficient oral delivery platform for antibacterial drugs, thereby achieving synergistic and amplified therapeutic efficiency. To further evaluate its drug-loading and release performance, the antibiotics drugs BER and NOR were incorporated into –SH/–S–S–@MSN *via* an extremely simple premixing process during gelling according to the solubility of the drug (Fig. 5A and B). Drug-loading capacities of BER–SH/–S–S–@MSN and NOR–SH/–S–S–@MSN were 4.49% and 4.42%, respectively (Fig. 5C). As confirmed by the FTIR and XRD spectra, BER/NOR–SH/–S–S–@MSN maintained the silica framework at 3643 cm^−1^ (*v* Si–O–Si), 1106.8 cm^−1^ (*v* Si–OH), and 473 cm^−1^ (*δ* Si–O–Si), along with the absence of fingerprint peaks and sharp peaks between 5° and 45° (Fig. 5D, E, and H). These data strongly verified that the drugs were highly dispersed and bound in the pore structure of –SH/–S–S–@MSN. In addition, the DSC thermograms of the drug free -HS/-S-S-@MSN were almost smooth lines with melting point depressions. After drug loading, the endothermic events of BER and NOR concerning intense and characteristic endothermic peaks at the melting points were diminished. Nitrogen adsorption-desorption tests also strongly illustrated the bound of BER and NOR into the nanopores on account of the degeneration of hysteresis loops in the isotherms (Fig. 5F and G). The stability of drug-loaded MSN after storage for 8 weeks at room temperature was further evaluated. No significant difference in either the aggregating profile in TEM or the peak shape in FTIR and XRD spectrum before and after storage was observed (Supporting Information Figs. S27 and S28). Furthermore, the chemical stability of the drug molecule during loading and release in different media was assessed. We compared the absorbance spectra of the bulk drug and the drug after loading and release, which revealed no significant changes (Supporting Information Figs. S29 and S30). Corresponding to the bioadhesion results, –SH/–S–S–@MSN significantly enhanced the retention of the loaded drug on the intestinal mucosa for 1‒1.2 times (Supporting Information Fig. S31).

As proposed, co-polymerization of –SH and –S–S– groups endows MSNs with dual-targeting capacity toward host ROS and GSH in inflammatory microenvironment and pathogenic bacteria, respectively. To be specific, the oxidation of –SH and –S–S– groups generates hydrophilic sulfoxide whereas reduction of –S–S– by intracellular GSH promoting the regeneration of –SH, thus facilitating the responsive and targeted drug release^35^. Thereafter, we quantified the drug release profile *via* the USP II paddle method, by using SIF containing H_2_O_2_ or GSH as the release media. It can be noted that –SH/–S–S–@MSN supported a sustained release pattern compared to the corresponding naked drugs, avoiding the degradation and rapid loss of the antibacterial agents, and alleviating the potential irritation and accumulative toxicity of the antibiotics. In addition, with the increasing concentration of H_2_O_2_ and GSH, the release of the drugs from –SH/–S–S–@MSN revealed a concentration-dependent manner, indicating its responsive capacity (Fig. 5J and K).

Subsequently, we thoroughly evaluated the bactericidal and bacteriostatic capacity of the drug-loaded –SH/–S–S–@MSN *in vitro* in comparison with the bulk drug. *Via* bacteria-MSNs co-culture, –SH modified MSNs significantly enhanced bacterial elimination compared to free BER or NOR, eradicating approximately 26% and 48% of the colony-forming units, respectively, which can be partially explained by the direct interaction between the –SH and the hydroxy group and mercaptan in bacterial membrane (Fig. 5L and M). Strikingly, the exposure of –SH/–S–S–@MSN achieved an approximately 75% bactericidal rate on account of the disruption of GSH balance. Similar trends were observed for NOR-loaded MSNs, due to the combination of the inherent antibacterial efficacy of antibiotics and the improved adhesion and responsiveness of MSNs. It is well noted that NOR–SH/–S–S–@MSN achieved100% bacteria elimination rate within 24 h, which was further confirmed by the OD600 growth curveOD_600_ curves after exposure to BER or NOR-loaded MSNs (Fig. 5O). The great affinity of –SH/–S–S–@MSN and BER/NOR–SH/–S–S–@MSN was further disclosed by the bio-TEM images, where the aggregated nanostructures gradually disintegrated into small cells which technically targeted around the peptidoglycan wall followed by cellular internalization, as mediated by the versatile –SH and –S–S– groups with balanced hydrophilicity (Fig. 5N). In summary, –SH/–S–S–@MSN with the capacity of mediating sustained release rendered the potential for improving the bioavailability and biosafety of conventional antibacterial drugs. Importantly, the functional –SH and –S–S– groups significantly enhanced the bactericidal efficiency due to the easily accessible action sites, concerning the hydroxide and mercaptan group in the bacterial membrane and the cytosol GSH balance, which was distinct from the traditional locus of antibiotics, *i.e.*, the nucleic acid within the nucleus.

### 3.7 Therapeutic efficacy of MSNs in bacterial enteritis in mice

**Figure 6:**
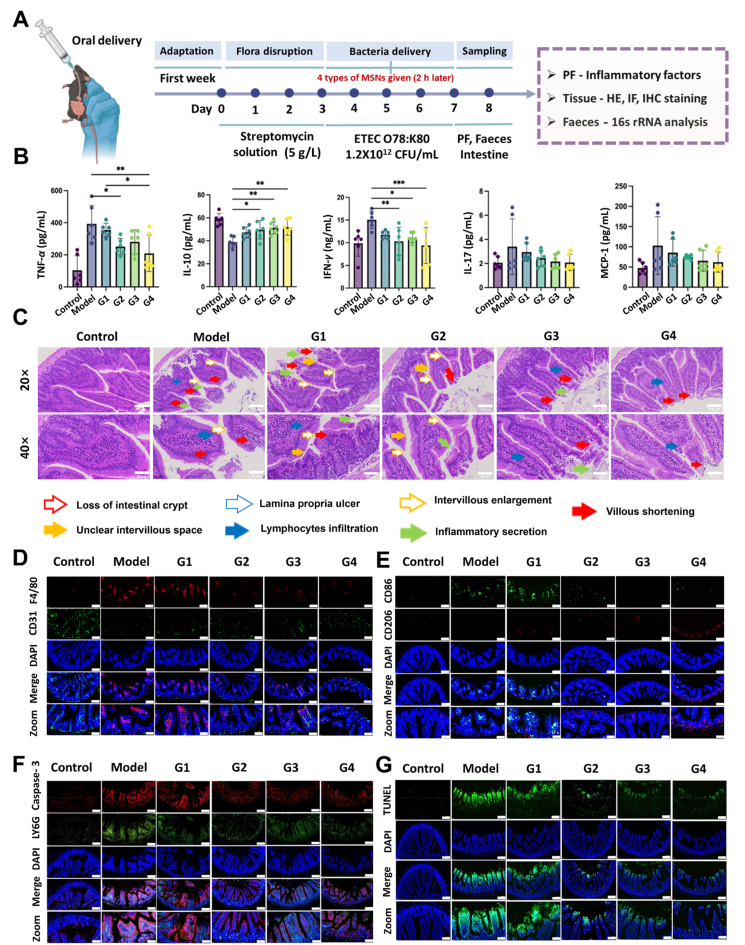
Therapeutic efficacyof –SH/–S–S–@MSN on bacterial enteritis. (A) Schematic illustration of the experimental procedures and the MSNs therapy strategy for bacterial enteritis in mice. (B) Cytokine levels in bacteria-infected mice before and after MSNs administration. (C) Hematoxylin-Eosin staining, scale bars = 100 μm (20 × magnification) and 50 μm (40 × magnification), respectively. (D) F4/80 and CD31 staining (blue: nucleistained with DAPI, green: endothelial cells, red: macrophages), scale bars = 100 μm (Normal) and 50 μm (Zoom), respectively. (E) CD86 and CD206staining (blue: nucleistained with DAPI, green: M1 macrophages, red: M2 macrophages), scale bar = 100 μm (Normal) and 50 μm (Zoom), respectively. (F) Caspase-3 and Ly6G staining (blue: nucleistained with DAPI, green: neutrophils, red: apoptotic cells, scale bars = 100 μm (Normal) and 50 μm (Zoom), respectively. (G) TUNEL staining (blue: nucleistained with DAPI, green: apoptotic cells) of small intestine tissues from bacteria infected mice before and after MSNs administration, scale bar = 100 μm (Normal) and 50 μm (Zoom), respectively. Data are presented as mean ± SD (*n* = 3), ∗*P* < 0.05, ∗∗*P* < 0.01, ∗∗∗*P* < 0.001, and ∗∗∗∗*P* < 0.0001 *vs*. indicated. A Created in BioRender. Li, H. (2025) https://BioRender.com/qg9kjtx.

**Figure 7:**
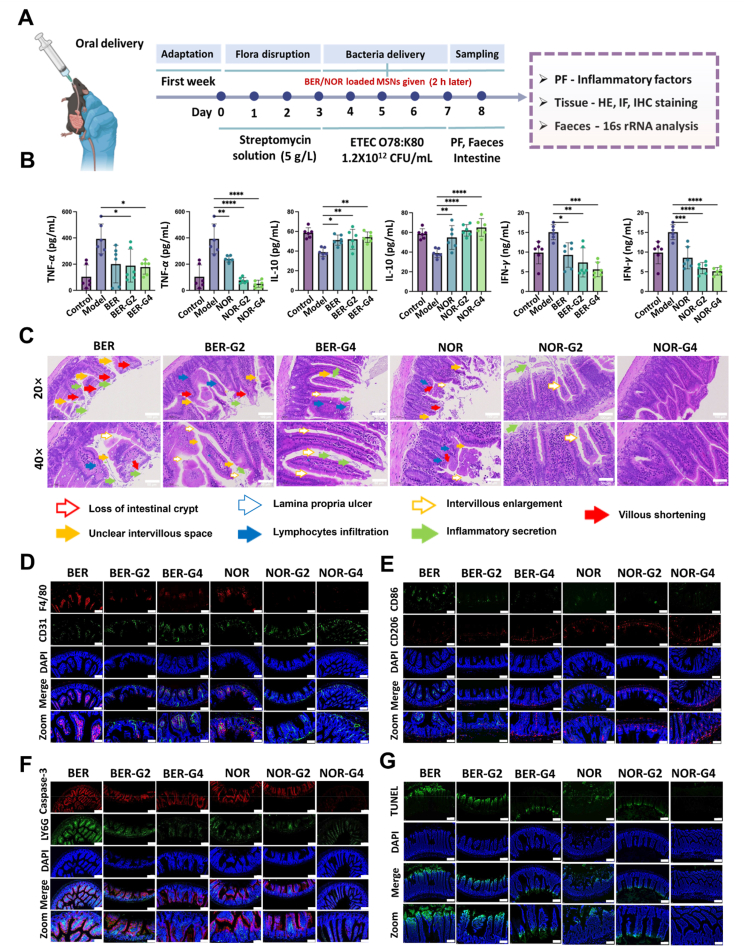
Therapeutic efficiency of BER/NOR–SH/–S–S–@MSN on bacterial enteritis. (A) Schematic illustration depicting the experimental procedures and the drug-loaded –SH/–S–S–@MSN therapy strategy for bacterial enteritis in mice. (B) Cytokine levels in bacteria-infected mice before and after BER/NOR–SH/–S–S–@MSN administration. (C) Hematoxylin-Eosin staining, scale bars = 100 μm (20 × magnification) and 50 μm (40 × magnification), respectively. (D) F4/80 and CD31 staining (blue: nuclei stained with DAPI, green: endothelial cells, red: macrophages), scale bar = 100 μm (Normal) and 50 μm (Zoom), respectively, (E) CD86 and CD206 staining (blue: nuclei stained with DAPI, green: M1 macrophages, red: M2 macrophages), scale bars = 100 μm (Normal) and 50 μm (Zoom), respectively. (F) Caspase-3 and Ly6G staining (blue: nuclei stained with DAPI, green: neutrophil, red: cell apoptosis), scale bars = 100 μm and 50 μm, respectively. (G) Tunel staining (blue: nucleistained with DAPI, green: apoptotic cells) of small intestine tissues from bacteria infected mice before and after BER/NOR–SH/–S–S–@MSN administration, scale bars = 100 μm (Normal) and 50 μm (Zoom), respectively. Data are presented as mean ± SD (*n* = 3), ∗*P* < 0.05, ∗∗*P* < 0.01, ∗∗∗*P* < 0.001, and ∗∗∗∗*P* < 0.0001 *vs*. indicated. A Created in BioRender. Li, H. (2025) https://BioRender.com/qg9kjtx.

Given the promising efficacy of –SH/–S–S–@MSN in eradicating etiologic pathogens and intestinal permeation, MSNs and BER/NOR-loaded MSNs were orally administered to bacteria-infected mice for therapy (Figure 6, Figure 7). In a typical run for inducing the pathological model, streptomycin solution was administered for 3 days to disrupt the flora homeostasis. Subsequently, ETEC O78:K80 at a concentration of 1.2 × 10^12^ CFU/mL was delivered for 4 days. To protect mice from bacterial infection, they were simultaneously orally given the MSNs, BER or NOR-loaded MSNs, and bulk drug (BER and NOR) in parallel with the bacteria given, alongside a control healthy group that received saline solution. On Day 8, the mice were sacrificed, and the intestine and faeces samples were collected for inflammatory cytokine quantification, hematein & eosin (HE), immunofluorescence (IF) staining, immunohistochemical (IHC) staining, Western blot (WB), 16S rRNA, and transcriptome analysis.

Within a week timeframe, healthy mice without bacterial infection showed normal performances, whereas the model mice experienced severe clinical symptoms of lassitude, emesis, and diarrhea with watery stools. After MSNs and antibacterial drugs (BER or NOR) therapy, the clinical symptoms were slightly relieved. It is worth noting that mice in the BER/NOR–SH/–S–S–@MSN groups showed normal performances without vomiting or diarrhea. Enzyme-linked immunosorbent assay (ELISA) suggested that –SH/–S–S–@MSN significantly restored intestinal homeostasis, reduced the levels of pro-inflammatory cytokines, including tumor necrosis factor-*α* (TNF-*α*), interleukin-1*β* (IL-1*β*), interlukine-6 (IL-6), interlukine-17 (IL-17), monocyte chemotactic protein-1 (MCP-1), myeloperoxidase (MPO) and interferon-*γ* (INF-*γ*), while upregulated the levels of anti-inflammatory cytokines including interleukin-4 (IL-4) and interleukin-10 (IL-10). Interestingly, –SH/–S–S–@MSN exhibited comparable capability with the free drug in reducing the inflammatory mediators, showing a great synergistic effect with BER or NOR after drug loading. As expected, the inflammatory mediators were further attenuated in the BER/NOR–SH/–S–S–@MSN groups (Figure 6, Figure 7B, Supporting Information Figs. S32 and S33).

H&E staining of the mice intestine tissues revealed hallmark features of bacterial enteritis in the model group, including loss of intestinal crypt, unclear intervillous space, lamina propria ulcer, intervillous enlargement, inflammatory secretion, villous shortening and infiltration of inflammatory cells (Fig. 6C). Compared with the MSNs groups, –SH/–S–S–@MSN significantly rescued the mice from bacterial infection on account of the less pathophysiological injury. Incorporation of the antibacterial drugs further enhanced the therapeutic outcomes, evidenced by the recovered normal mucosal structure, especially in the BER/NOR–SH/–S–S–@MSN group with almost normal intestinal microstructures (Fig. 7C). Furthermore, IF studies were carried out to reveal the immune microenvironment and cellular phenotype. F4/80 and CD31 staining indicated macrophage activation and endothelial cell damage in mice with bacterial enteritis. With the assistance of MSNs, the damage to the epithelium and the infiltration of macrophages were relieved. Especially in mice treated with BER/NOR–SH/–S–S–@MSN, the cellular morphology returned to normal (Figure 6, Figure 7D, Supporting Information Figs. S34 and S35). Meanwhile, the IF staining concerning to macrophages polarization showed that the CD86 expression (assigned to M1 macrophages) was significantly upregulated in the intestine of bacterial enteritis mice, while treatment with –SH/–S–S–@MSN promoted a phenotypic shift toward M2 macrophages, indicating of an anti-inflammatory response (Figure 6, Figure 7E, Figs. S34 and S35). Neutrophil activation f (Ly6G) and apoptosis (cleaved caspase 3) were substantially alleviated in BER/NOR–SH/–S–S–@MSN groups, reaching levels comparable to the healthy control (Figure 6, Figure 7F, Figs. S34 and S35). Additionally, TUNEL staining revealed that apoptosis was also alleviated by the proposed nanostructures (Figure 6, Figure 7G, Figs. S34 and S35).

### 3.8 Therapeutic mechanism of MSNs in bacterial enteritis mice

**Figure 8:**
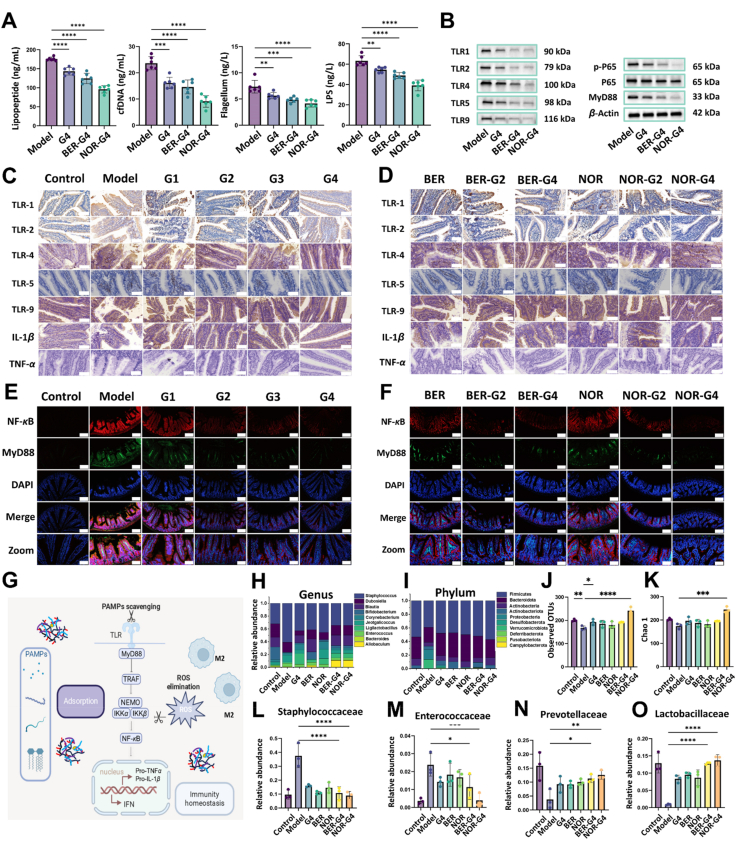
Molecular mechanism of BER/NOR–SH/–S–S–@MSN on bacterial enteritis treatment. (A) Level of lipopeptide, cfDNA, LPS, and flagellum *in vivo* before and after –SH/–S–S–@MSN or BER/NOR–SH/–S–S–@MSN treatment. (B) WB analysis of MyD88, P65, p-P65, TLR-1, TLR-2, TLR-4, TLR-5, TLR-9, and *β*-actin. IHC staining (corresponding toTLR1, TLR2, TLR4, TLR5, TLR9, TNF-*α* and IL-1*β* expressions) before and after administrating. (C) MSNs, scale bar = 10 μm and (D) BER/NOR–SH/–S–S–@MSN. NF-*κ*B and MyD88 staining (blue: nucleus stained with DAPI, green: MyD88, red: NF-*κ*B) of small intestine tissues from bacteria-infected mice before and after administering (E) MSNs and (F) BER/NOR–SH/–S–S–@MSN, scale bars = 200 μm and 100 μm. (G) Schematic illustration of the molecular mechanism of –SH/–S–S–@MSN or BER/NOR loaded –SH/–S–S–@MSN on reprogramming the inflammatory microenvironment of bacterial enteritis. 16s rRNA sequencing analysis containing percentages of community abundance on the (H) genus and (I) phylum level, observed (J) OTUs, and (K) Chao 1 index. Relative abundance of specific gut bacteria, including (L) *Staphylococcaceae*, (M) *Enterococcaceae*, (N) *Prevotellaceae*, and (O) *Lactobacillaceae*, before and after MSNs and BER/NO-–SH/–S–S–@MSN treatment. Data are presented as mean ± SD, *n* = 3, ∗*P* < 0.05, ∗∗*P* < 0.01, ∗∗∗*P* < 0.001, and ∗∗∗∗*P* < 0.0001 *vs*. indicated. G Created in BioRender. Li, H. (2025) https://BioRender.com/qg9kjtx.

To our knowledge, TLRs can recognize specific PAMPs or DAMPs to drive inflammatory immunity during bacterial infection, which play crucial roles in the development of bacterial enteritis and promote a vicious inflammatory cycle. To be specific, subcellular fractions of *E. coli*, such as lipopeptides, lipopolysaccharides, and flagellacan activate the TLR1/2, TLR4, and TLR5, simultaneously. TLR9 can recognize microbial or host cfDNA. Considering the pathogen elimination and adjustment functions of MSNs, we further studied the expression of TLR 1, 2, 4, 5, 9 in bacterial enteritis mice before and after MSNs and BER or NOR-loaded MSNs treatments. IHC images validated the activation of TLR1, 2, 4, 5, 9 in bacterial enteritis mice on account of their recognition of pathogenic bacteria and the metabolites, along with the elevated expression of MyD88 and NF-*κ*B than the untreated normal mice (Fig. 8C–F, Supporting Information Figs. S36–S38). Owing to the –SH/–S–S–@MSN and BER/NOR–SH/–S–S–@MSN treatment, the activation of bacterial lipopeptide, LPS, flagellum, pathogen-released DNA, and host cfDNA was effectively controlled and eliminated. We collected the peritoneal solution from C57BL/6 mice in the model, –SH/–S–S–@MSN, and BER/NOR–SH/–S–S–@MSN delivered groups, which were utilized for assessing the LPS, lipopeptide, and flagella and cfDNA *in vivo*. Research shows that after MSN treatment, the levels of four main pathogenic mediators significantly decreased by 32.25%, 44.2%, 42.9%, and 62.5%, respectively (Fig. 8A and G). As expected, they also reduced the TLRs activation as well as the downstream release of inflammatory factors (including TNF-*α* and IL-1*β*) in mice, thus attenuating the immune response as expected (Fig. 8C and D). Furthermore, Western blot (WB) analysis for TLR 1, 2, 4, 5, 9 and downstream p65/p-P65 and MyD88 mediators involved in inflammatory signaling was thoroughly carried out. After –SH/–S–S–@MSN, BER/NOR–SH/–S–S–@MSN treatment, the relative expression of the associated proteins was significantly decreased, which efficiently reflects the interaction between MSN and pro-inflammatory subcellular factors *in vivo* (Fig. 8A and B, Supporting Information Figs. S39 and S40). In addition, transcriptomics analysis on the intestine tissues of mice was conducted to reveal the therapeutic effect of NOR–SH/–S–S–@MSN at the gene level. In comparison with the control group, NOR–SH/–S–S–@MSN downregulated the expression of *Fcmr*, *Sell*, *Serpina3i*, *Blk*, *Pax5*, *Ms4a1*, *Cr2*, *Cxcr5*, *Ccr6*, *Cd19*, *Grm6*, *Chsr3*, *Siglecg gene*. A differential gene cluster heatmap indicated that NOR–SH/–S–S–@MSN therapy significantly changed gene expression related to cellular processes, environmental information processing, and human diseases. Kyoto Encyclopedia of Genes and Genomes (KEGG) analysis highlighted differentially expressed genes in the phagosome, NF-*κ*B signaling pathway, and cytokine–cytokine receptor interaction, which are highly associated with the inflammatory cascade and the secretion of pro-inflammatory cytokines related to the pathogenesis and disease progression of bacterial enteritis (Supporting Information Figs. S41 and S42).

Finally, fecal samples of mice in each group were collected on Day 7 for 16S rRNA sequencing to assess the gut microbiome composition. *E. coli* infection markedly reduced in microbial diversity and community richness, as indicated by *α*-diversity metrics (Chao1 and Observed OTUs). It is worth noting that treatment with –SH/–S–S–@MSN and BER/NOR–SH/–S–S–@MSN significantly restored microbiome diversity and abundance (Fig. 8J and K). Taxonomic analysis at the phylum and genus levels demonstrated that ETEC infection resulted in an overrepresentation of pathogenic *Deferribacterota*, *Desulfobacteriota*, *Proteobacteria*, *Staphylococcus*, and *Enterococcus*, while beneficial taxa such as *Firmicutes, Bacteroidota, Actinobacteria, Allobaculum,* and *Bacteroides* were depleted (Fig. 8H and I). Notably, BER/NOR–SH/–S–S–@MSN administration reversed the dysbiosis by significantly enriching Lactobacillaceae, which facilitated polysaccharide metabolism and probiotic proliferation, as well as Prevotellaceae, as a key cornerstone of intestinal homeostasis (Fig. 8L–O)^36^^,^^37^. Collectively, these findings demonstrated that both –SH/–S–S–@MSN and drug-loaded –SH/–S–S–@MSN effectively reconstituted gut-microbial homeostasis, suppressed pro-inflammatory signaling, and enhanced the abundance of commensal bacteria while reducing pathogenic colonization. These multifaceted therapeutic benefits underscore the potential of the multivalent sulphur-modified biosilica nanostructures as a promising intervention for bacterial enteritis.

## 4 Conclusions

Bacterial enteritis remains a significant global health concern, particularly affecting vulnerable populations such as neonates and immunocompromised individuals. Conventional antibacterial drugs often fall short in infection control and drive rising antimicrobial resistance, owing to their poor management, drug delivery, and *in vivo* fate, and inability to regulate immune regulation and microbiota remodeling. In this study, we developed a novel –SH/–S–S–@MSN with PEI template, silica network, and multivalent sulphur-modification to simultaneously eradicate the pathogenic bacteria, regulate the immune response, and reprogram the inflammatory microenvironment in the infected intestine. Our findings demonstrated that the nanostructure not only eradicated pathogenic bacteria but also modulated oxidative stress, attenuated inflammation, and promoted gut microbiota restoration. Referring to the multivalent sulphur-modification, the –S–S– with oxidability perturbed the GSH balance within the bacteria, while the combined reductive capacity of –SH and –S–S– scavenged ROS and mitigated inflammation-induced damage. The nanopores with the positively charged PEI facilitated the absorption of various inflammatory mediators, including bacterial LPS, lipopeptide, flagellum, and bacterial/host cfDNA through hydrogen bonding and electrostatic interaction. Furthermore, –SH/–S–S–@MSN with appropriate surface/interface properties achieved the long-term intestinal retention and deep permeation, significantly reduced pro-inflammatory cytokine secretion, facilitated M2 macrophage polarization, and mitigated epithelial injury, thereby reprogramming the inflammatory microenvironment. Moreover, the incorporation of conventional antibiotics such as NOR and BER further enhanced therapeutic efficacy while minimizing systemic toxicity. Gut-microbiota analysis further confirmed that treatment with –SH/–S–S–@MSN and BER/NOR–SH/–S–S–@MSN effectively suppressed pathogenic bacterial populations while promoting the proliferation of beneficial commensal bacteria, thereby restoring microbial equilibrium and reinforcing intestinal barrier integrity. By integrating the flexible REDOX responsiveness excellent biocompatibility, intestine-targeted delivery, and ease of fabrication, the innovative nanostructure offers a promising next-generation antimicrobial strategy with reduced resistance potential and improved clinical outcomes.

## Acknowledgments

This work was supported by the Scientific Project of Liaoning Provincial Science and Technology Program Projects (No. 2024-MS-029 [Jaili Han], China), the 10.13039/501100001809National Natural Science Foundation of China (No. 82472136 [Heran Li], China), Liaoning Provincial Youth Science Foundation, Category B (No. 2025-YQ-10 [Heran Li], China), and Scientific Research Innovation Capability Support Project For Young Faculty (SRICSPYF-BS2025082 [Heran, Li], China). The graphical abstract figure was created in BioRender. Li, H. (2025) https://BioRender.com/ag4homw.

## Author contributions

Heran Li, Jiali Han, Xuting Zheng and Long He designed the research. Tongyi Zhao and Miao Xu carried out the experiments and performed data analysis. Yuman Sun, Jiatong Li, Jingjie Sun, and Ziwei Hao participated part of the experiments. Xiaoxi Fan and Haijia Hou provided experimental drugs and quality control. Tongyi Zhao wrote the manuscript. Heran Li revised the manuscript. All of the authors have read and approved the final manuscript.

## Conflicts of interest

The authors declare no conflicts of interest.

## Appendix A Supporting information

The following is the Supporting Information to this article.
