# Supplementary material for: Multivalent sulphur-modified biosilica nanostructures for bacterial enteritis therapy
Source: Acta Pharm Sin B. 2026 Jan 1;16(3):1643–61. doi: 10.1016/j.apsb.2025.12.045 (PMC13031151; doi:10.1016/j.apsb.2025.12.045)
Supplement: Multimedia component 1 [file mmc1.pdf]

Original article

Multivalent sulphur-modified biosilica nanostructures for bacterial enteritis therapy

Tongyi Zhao[a,b,†], Xiaoxi Fan[c,†], Haijia Hou[d,†], Miao Xu[a], Yuman Sun[a], Jingjie Sun[a], Ziwei Hao[a], Xuchun Chen[e], Long He[f,*], Xuting Zheng[g,*], Heran Li[a,*], Jiali Han[b,*]

[a]*School of Pharmacy, China Medical University, Shenyang 110122, China*

[b]*Department of Otorhinolaryngology, The First Hospital of China Medical University, Shenyang 110001, China*

[c]*Department of Thoracic Surgery, The First Hospital of China Medical University, Shenyang 110001, China*

[d]*Department of Respiratory and Critical Care Medicine, The First Hospital of China Medical University, Shenyang 110001, China*

[e]*Department of Organ transplantation and Hepatobiliary surgery, The First Hospital of China Medical University, Shenyang 110001, China*

[f]*Organ transplantation center, General Hospital of Northern Theater Command, Shenyang 110010, China*

[g]*Department of Infectious Disease, The First Hospital of China Medical University, Shenyang 110001, China*

Received 19 June 2025; received in revised form 7 September 2025; accepted 29 September 2025

*Corresponding authors.

E-mail addresses: helongjimmie@163.com (Long He), zxtskycs@163.com (Xuting Zheng), liheranmm@163.com (Heran Li), Jiali_han@163.com (Jiali Han).

[†]These authors made equal contributions to this work.

Supporting Figures

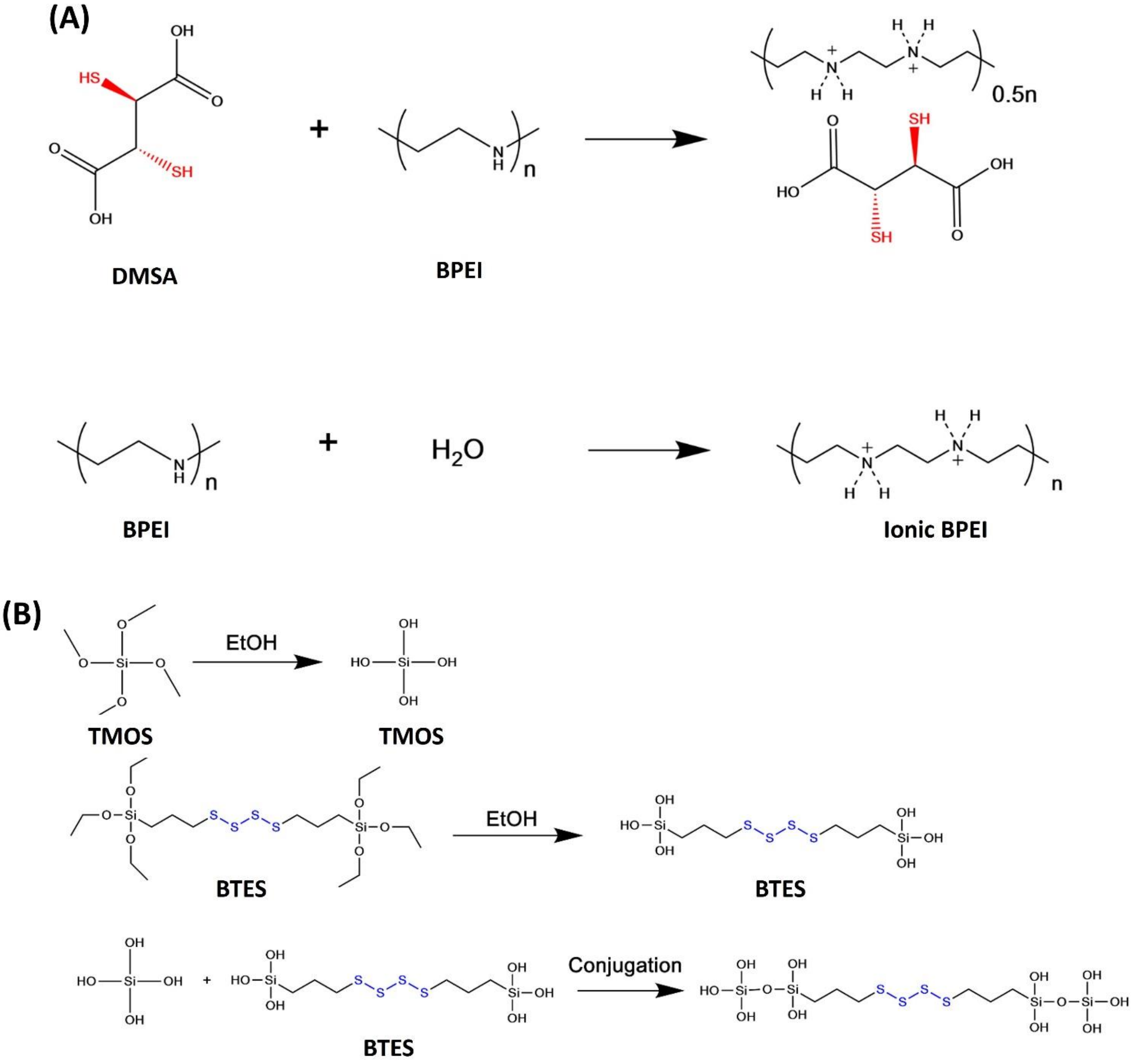

Figure S1 The biomimetic synthesizing strategies of four types of MSNs in 2D format. (A) Aqueous phase reaction. (B) Organic phase reaction.

(A) G1

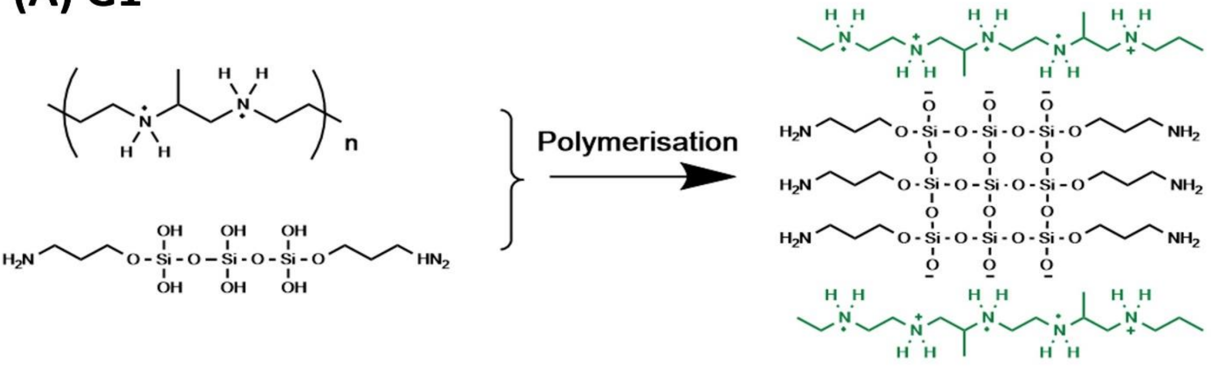

(B) G2

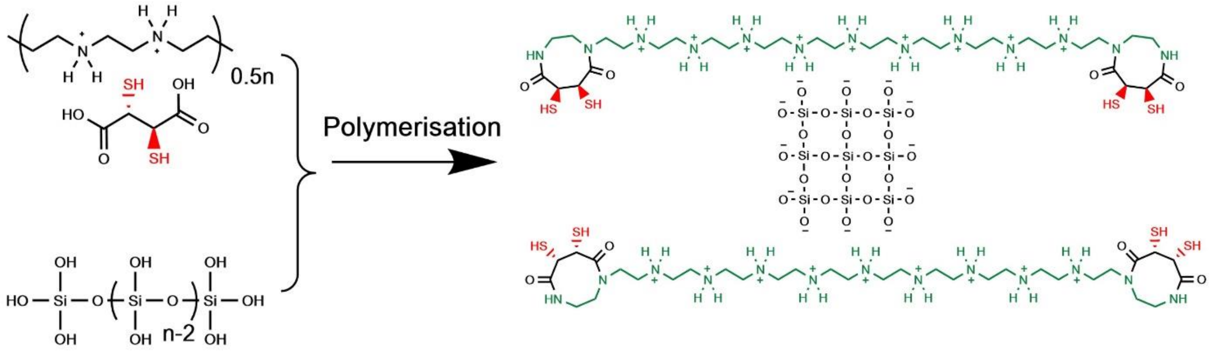

(C) G3

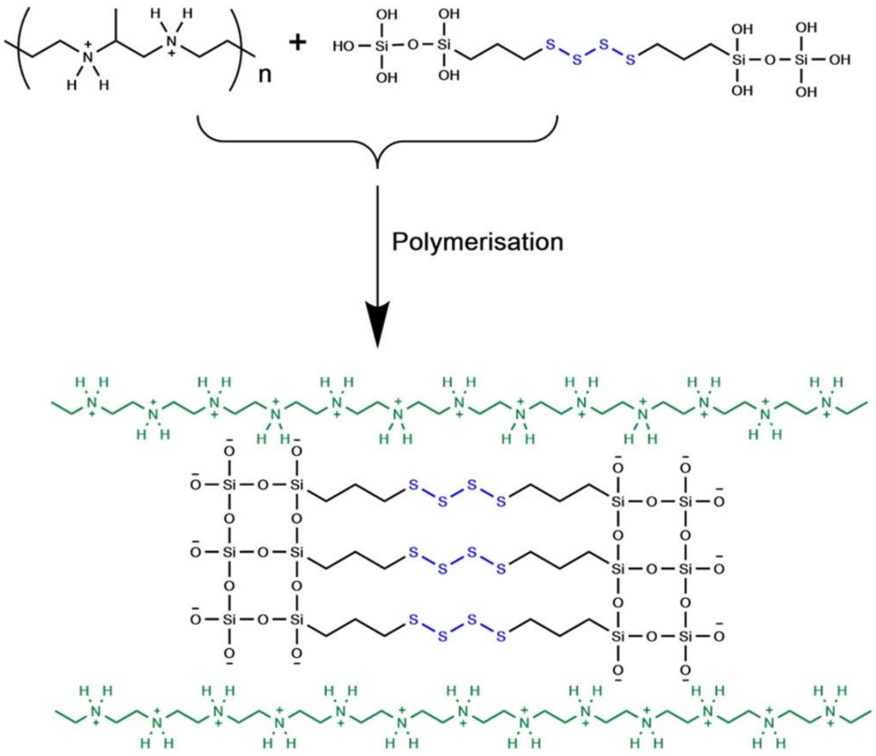

(D) G4

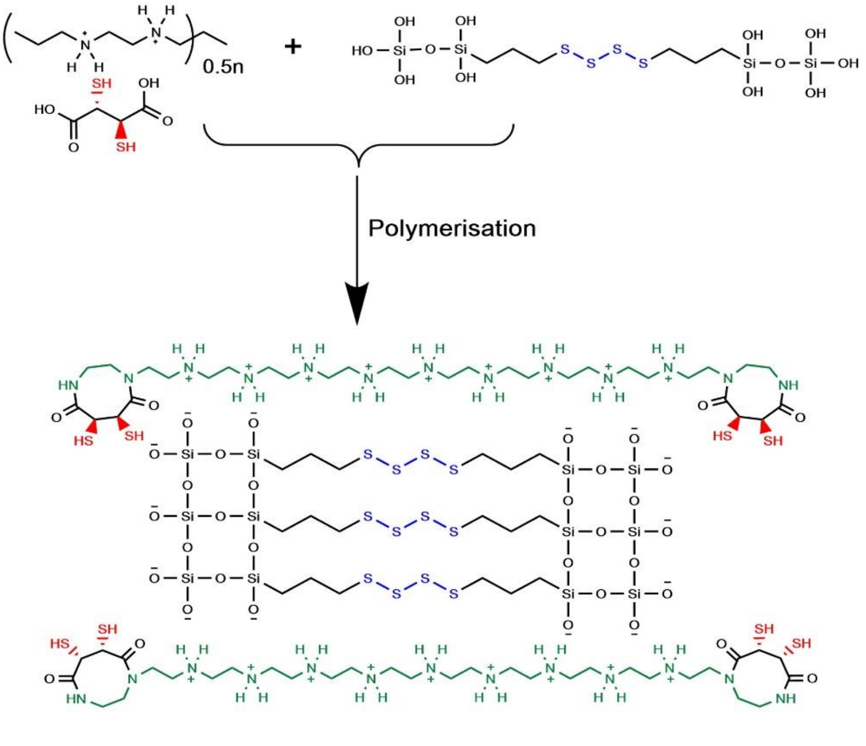

Figure S2 The biomimetic polymerization process of the final biosilica nanostructure. (A) G1, (B) G2, (C) G3 and (D) G4.

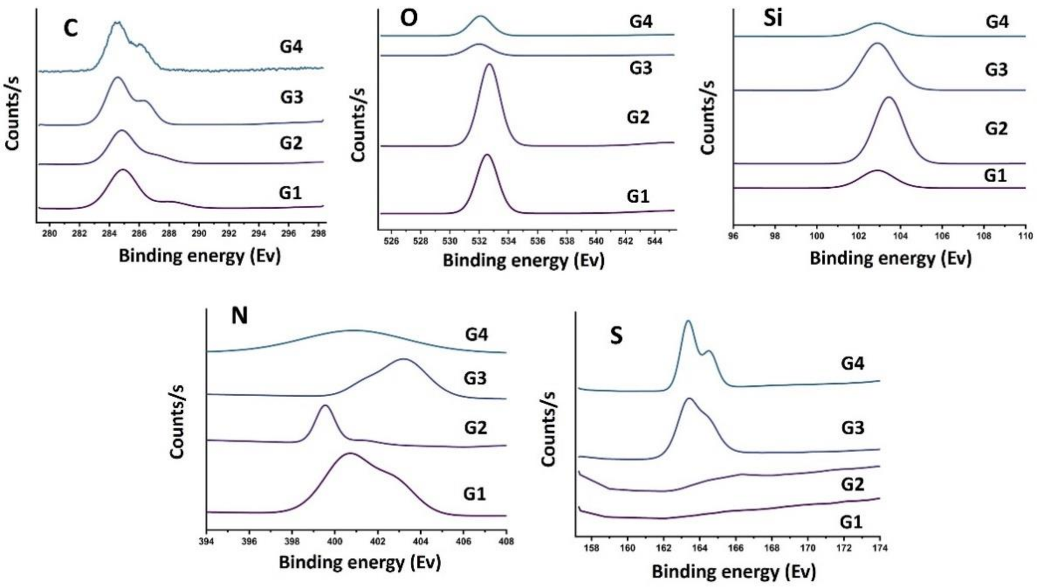

Figure S3 XPS spectra of four types of MSNs.

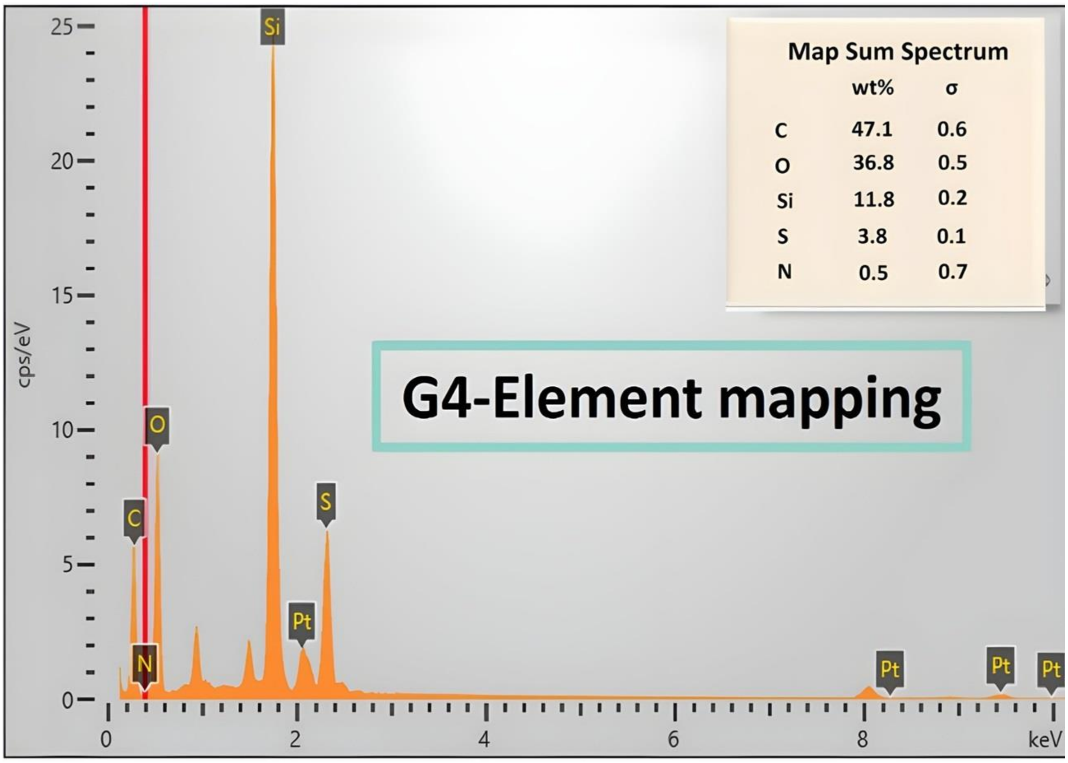

Figure S4 EDS mapping of G4.

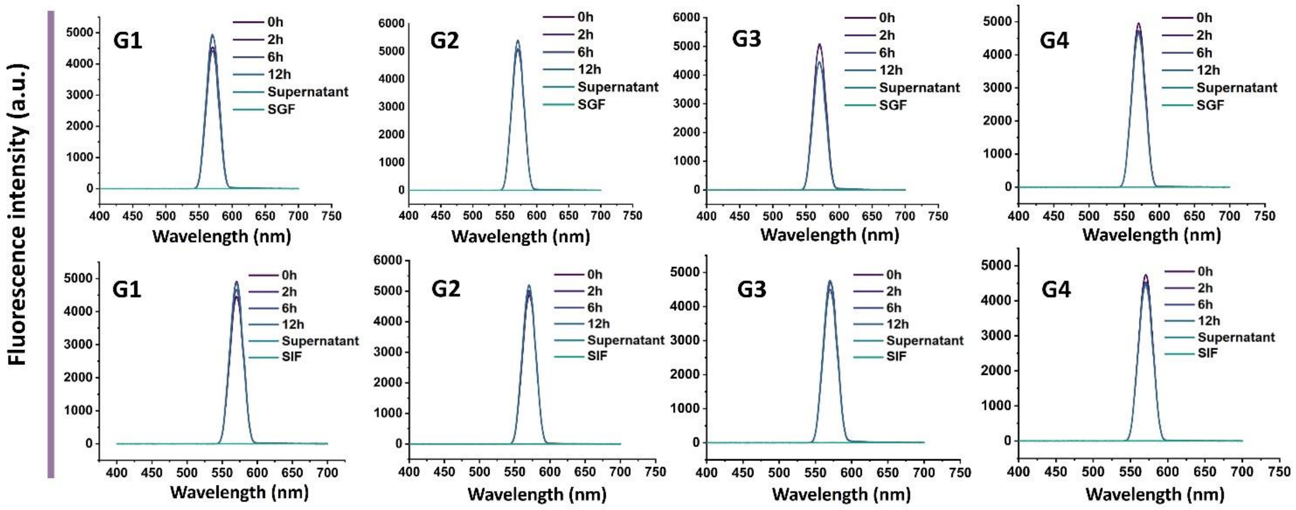

Figure S5 The fluorescence stability of RITC-labeled MSNs in SGF and SIF.

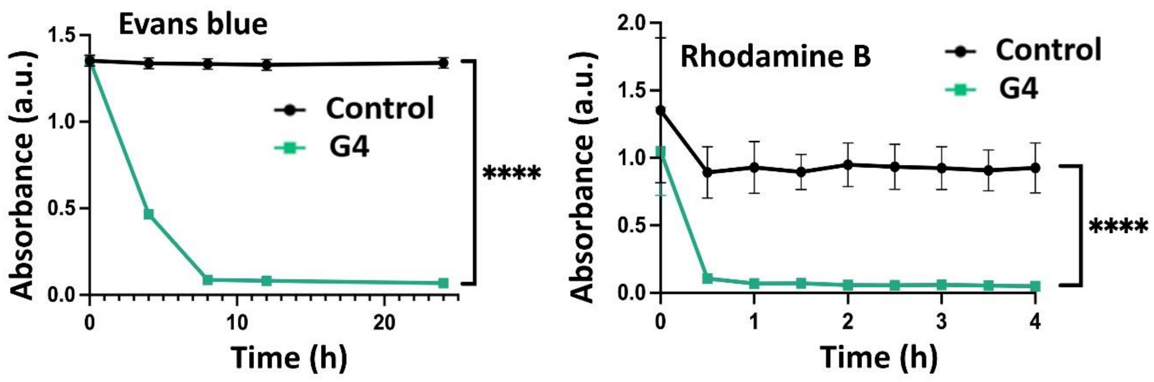

Figure S6 Absorption capacity of G4 for Rhodamine B and Evans blue. Data are presented as mean ± SD (n = 3), *P < 0.05, **P < 0.01, ***P < 0.001, and ****P < 0.0001 *vs*. indicated.

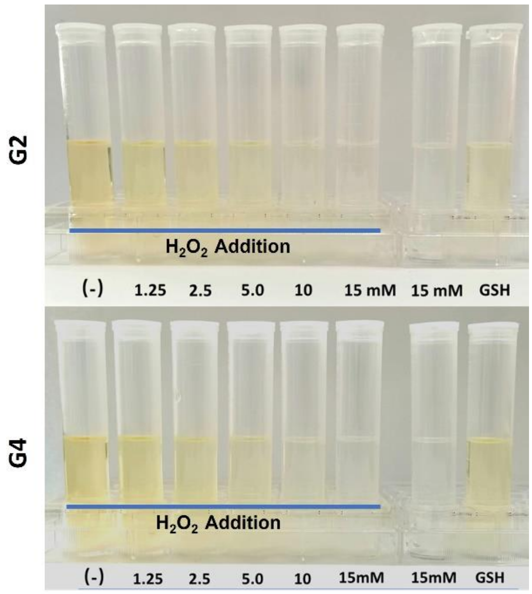

Figure S7 DTNB analysis for detecting the existence of –SH groups in G2 and G4.

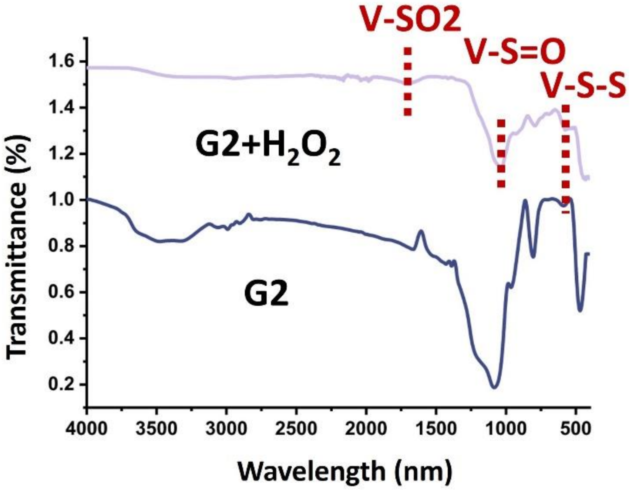

Figure S8 FTIR spectrum of G2 before and after incubation with H_2O_2.

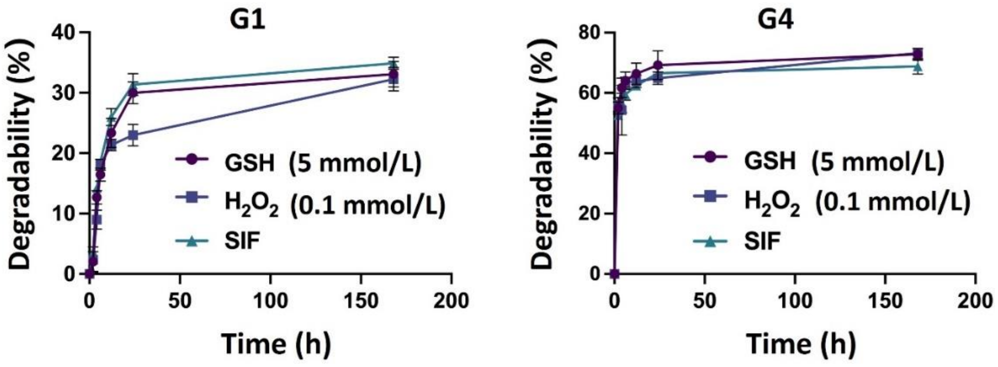

Figure S9 The degradation profiles of G1 and G4 in various media concerning SIF as well as SIF containing GSH and H_2O_2. Data are presented as mean ± SD (n = 3).

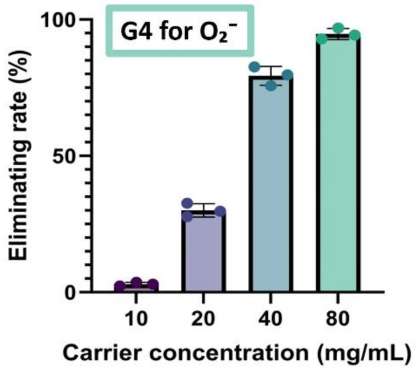

Figure S10 Concentration-dependent O_2^- elimination profile of G4. Data are presented as mean ± SD (n = 3).

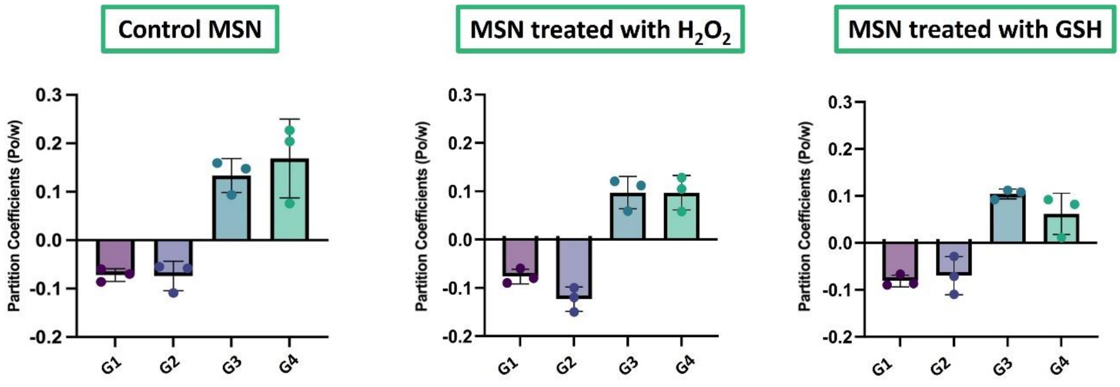

Figure S11 Oil/water partition coefficients of G4 before and after treating with H_2O_2 and GSH. Data are presented as mean ± SD (n = 3).

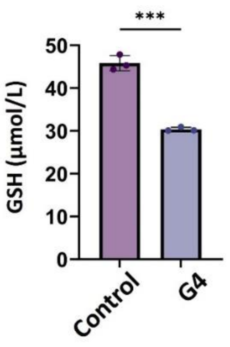

Figure S12 The change of GSH concentration within the bacteria before and after G4 treatment. Data are presented as mean ± SD (n = 3), ***P < 0.001 *vs*. indicated.

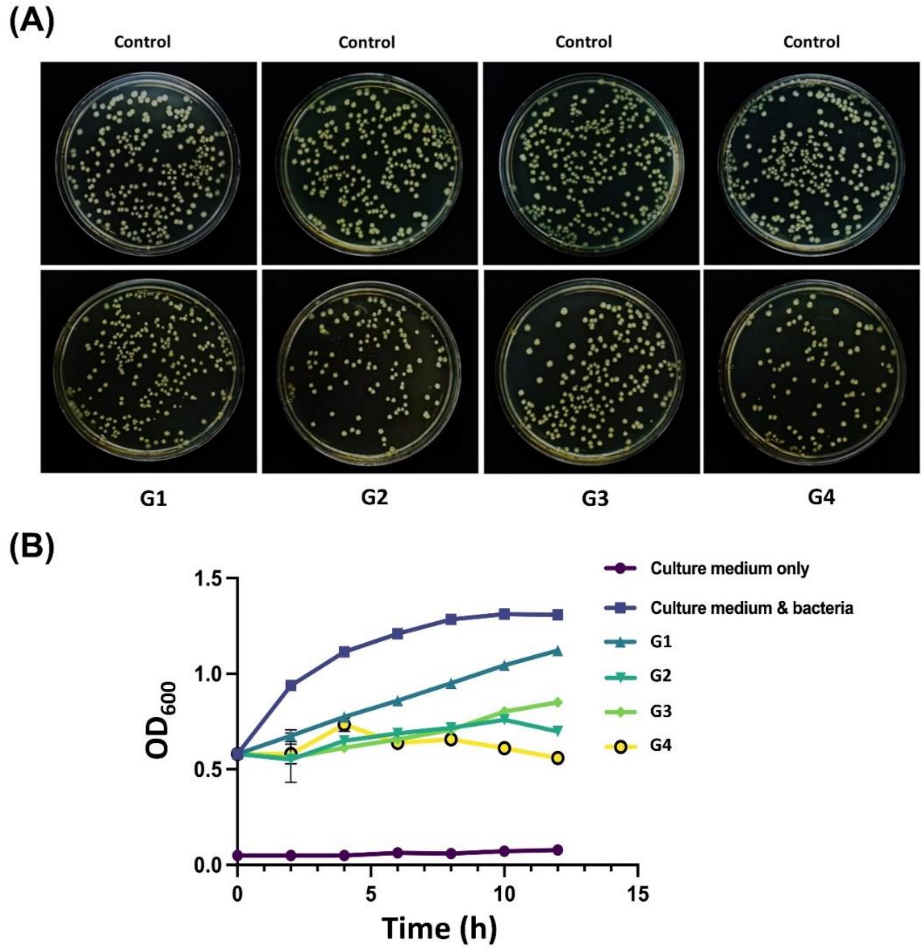

Figure S13 (A) Images of the *in vitro* anti-bacteria profiles of four types of blank MSNs. (B) OD_{600} growth curve of normal *E. coli*, and those cocultured with MSNs. Data are presented as mean ± SD (n = 3).

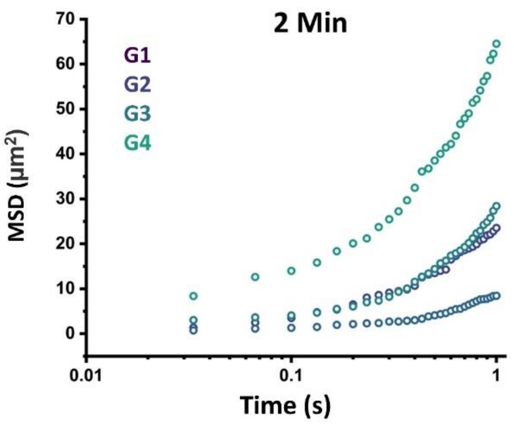

Figure S14 MSD of four types of MSNs within 2 min.

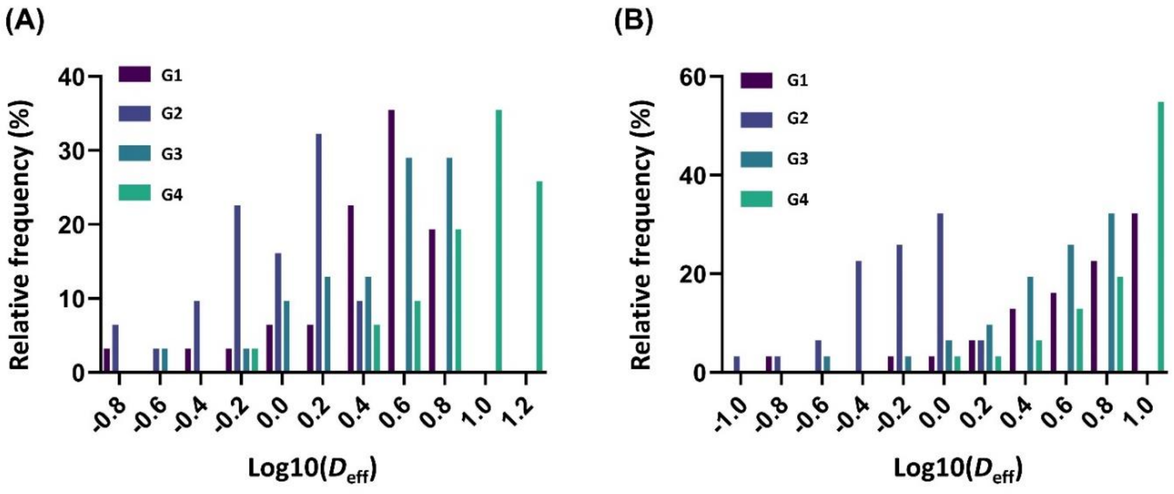

Figure S15 Distributions on the logarithms of effective diffusivities (D_{eff}) of MSNs penetrated in the healthy mice mucus at a time scale of (A) 2 min and (B) 5 min.

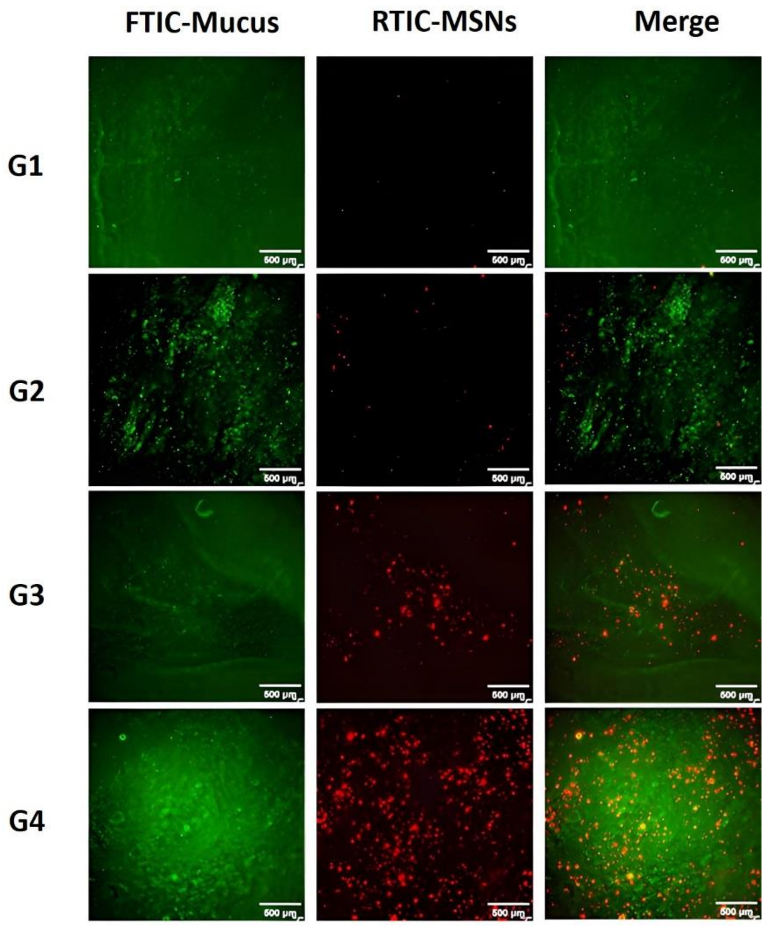

Figure S16 2D coverage of RITC labeled MSNs (red) diffusing in the rat intestinal mucus stained with FITC (green) *ex vivo* acquired by CLSM. Scale bar=500 μm.

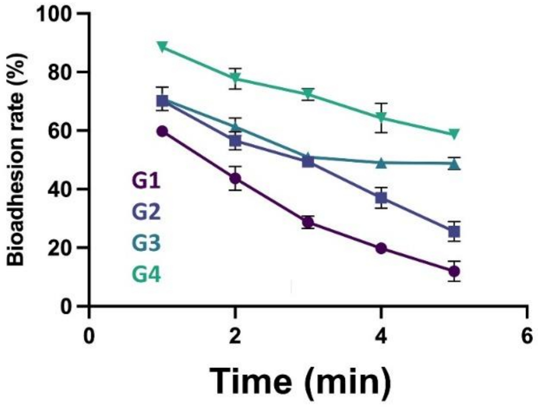

Figure S17 The bioadhesion profiles of MSNs on the rat small intestine. Data are presented as mean ± SD (n = 3).

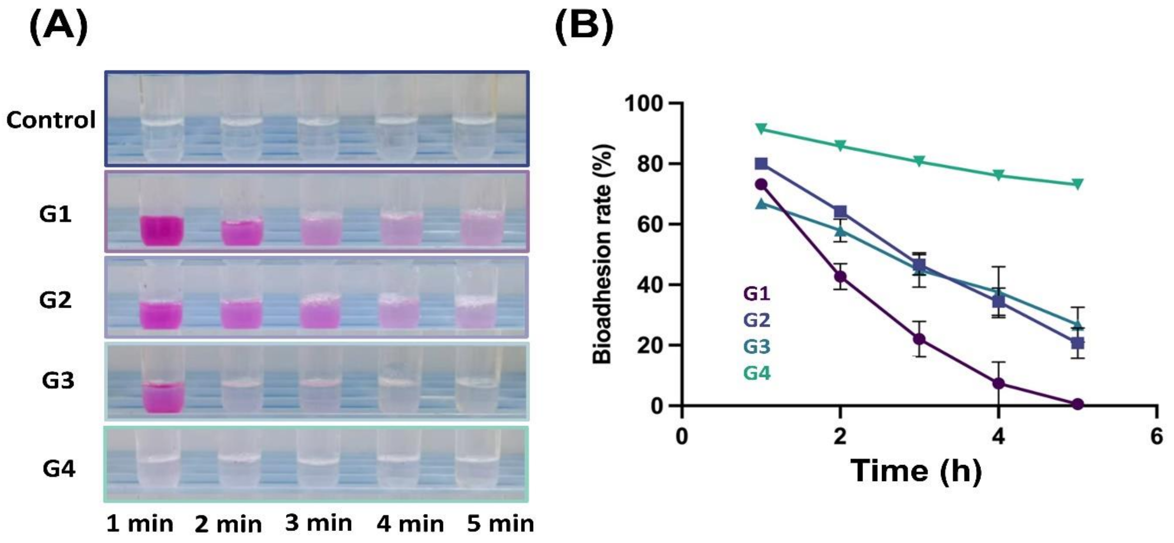

Figure S18 The (A) image and (B) quantitative analysis of the bioadhesion capacities of RITC labeled MSNs on the rat intestinal tissues under the elution of SIF. Data are presented as means ± SD (n = 3).

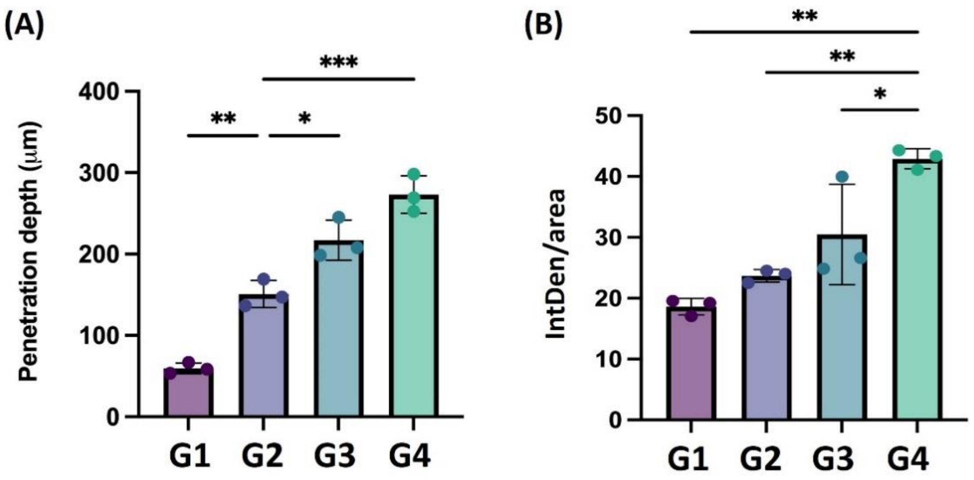

Figure S19 Penetrationdepth (A) and fluorescence intensity (B) of MSNs penetrated across the intestinal mucosa in the ex vivo intestinal adhesion study. Data are presented as mean ± SD, $n = 3$, *$P < 0.05$, **$P < 0.01$, and ***$P < 0.001$.

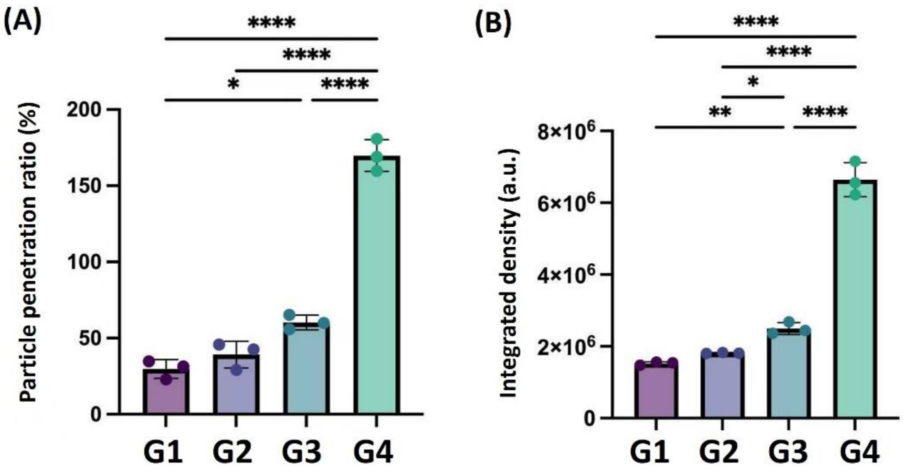

Figure S20 Penetration depth and fluorescence intensity of MSNs penetrated across the intestinal mucosa *in vivo*. Data are presented as mean ± SD, $n = 3$, *$P < 0.05$, **$P < 0.01$, and ****$P < 0.0001$ *vs*. indicated.

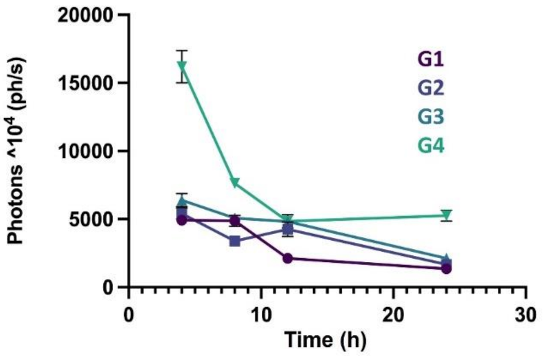

Figure S21 The semi-quantification of fluorescence intensity of RITC labeled nanomedicines in the GIT after oral administration. Data are presented as means ± SD (*n* = 3).

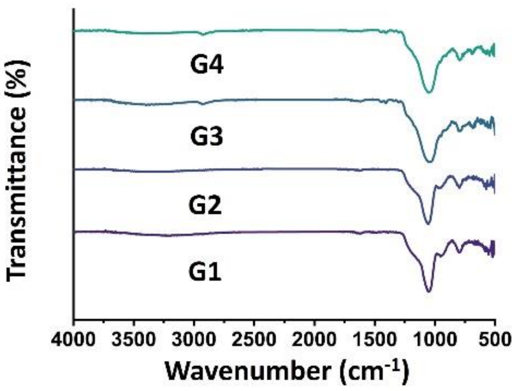

Figure S22 The stability of MSNs within gastric acid (pH 1.0). FTIR spectrum of MSNs after immersing in pH 1.0 hydrochloric acid for 2 h.

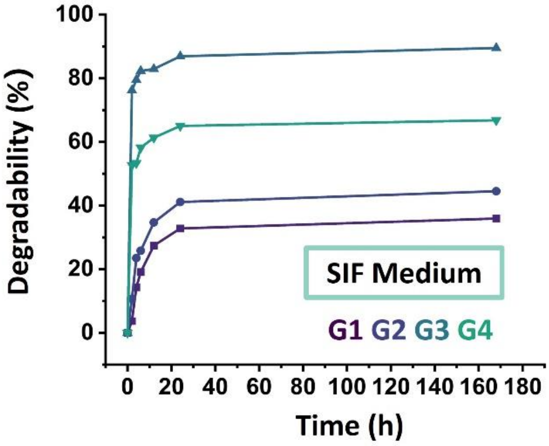

Figure S23 The degradation profiles of four MSNs within the SIF medium for 7 days.

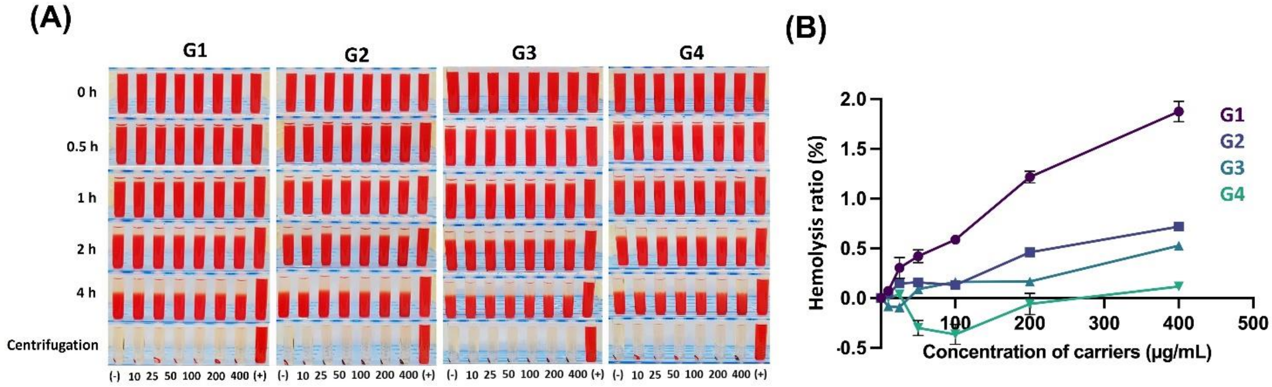

Figure S24 (A) Blood sedimentation images and (B) the hemolysis ratio of MSNs (n=3).

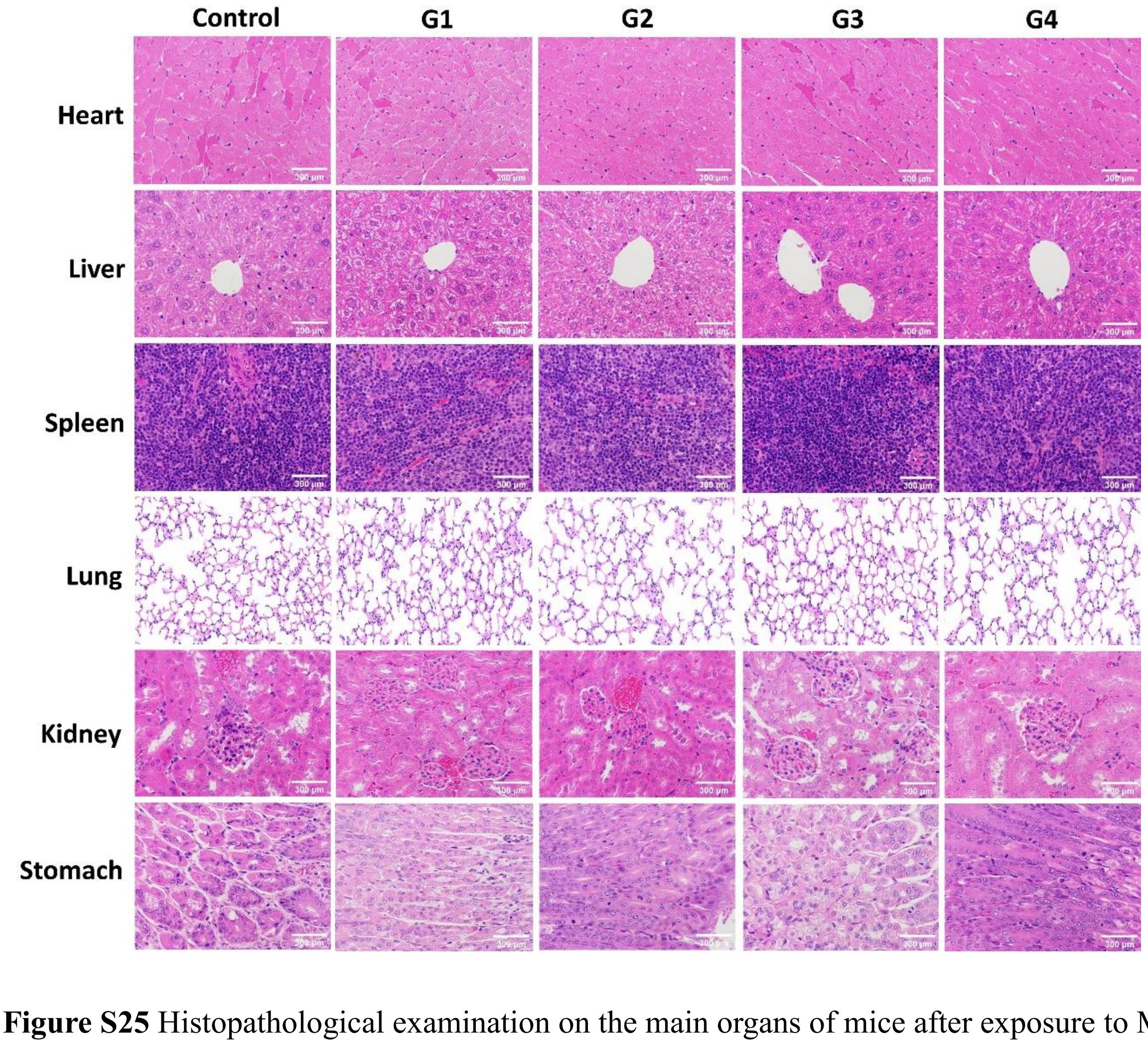

Figure S25 Histopathological examination on the main organs of mice after exposure to MSNs for 14 days (n=3). Scale bar=300 μm.

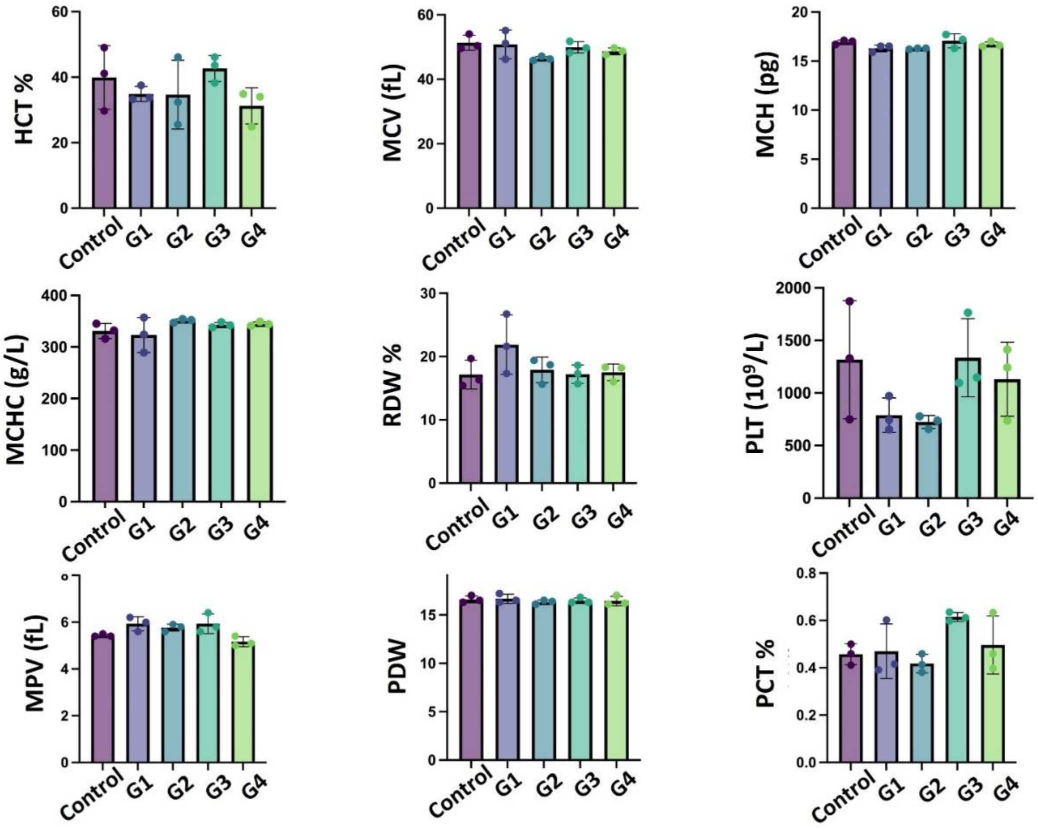

Figure S26 Hematology indexes of mice were measured after exposure to MSNs for 14 days. Data are presented as mean ± SD (*n* = 3).

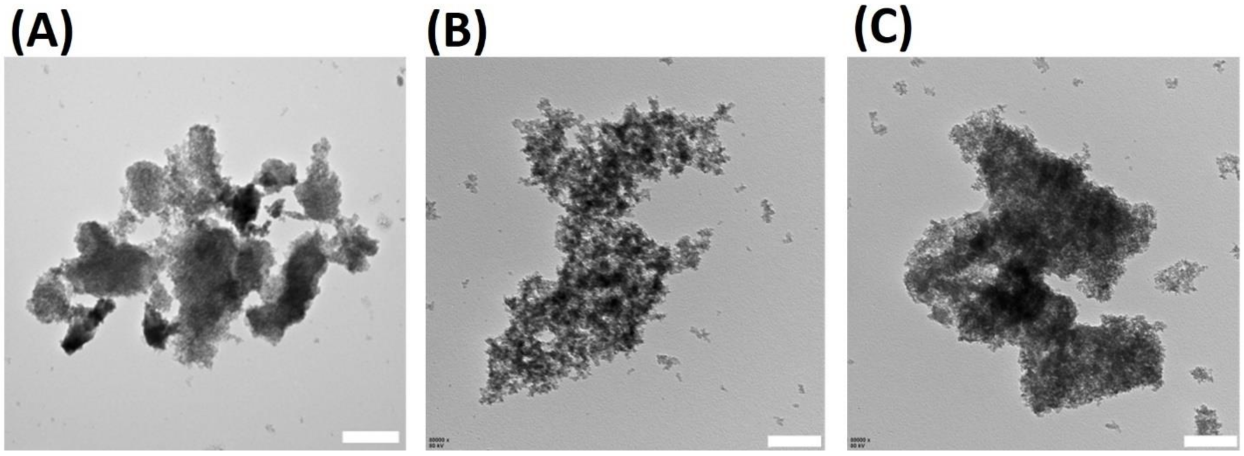

Figure S27 The TEM images of (A) G4, (B) BER-G4, and (C) NOR-G4 were taken after storage for 8 weeks. Scale bar=200 nm.

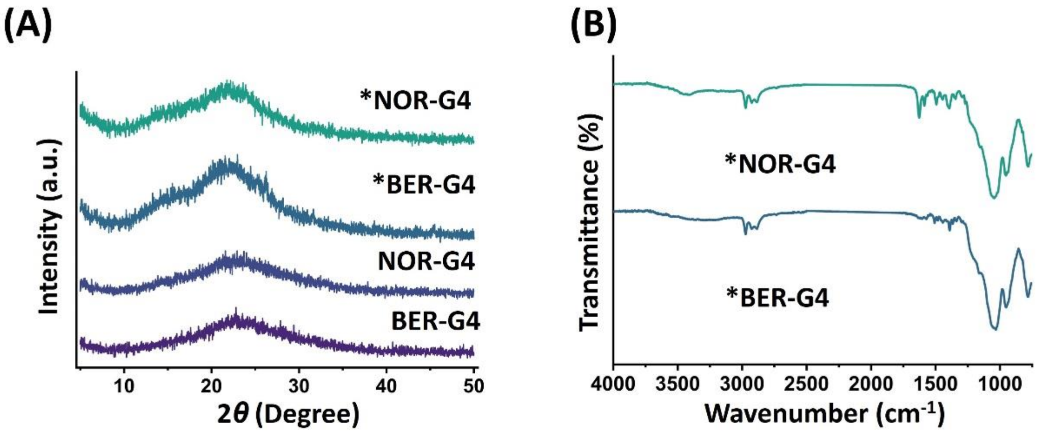

Figure S28 (A) XRD patterns and (B) FTIR spectra of BER/NOR-G4; the asterisk (*) marks the sample after 8-week storage.

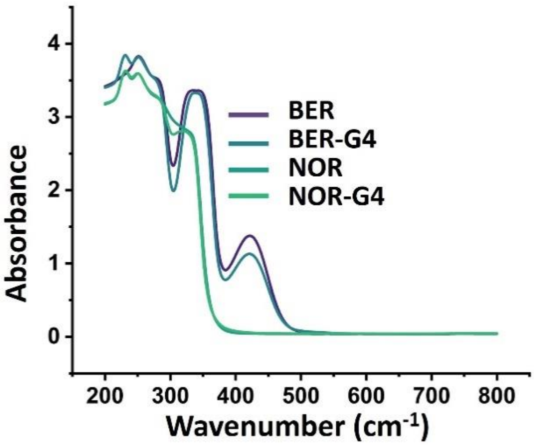

Figure S29 UV–Vis absorption spectra showing the stability of BER/NOR which were loaded into G4 followed by being released into the SIF (pH 6.8, 37 ℃).

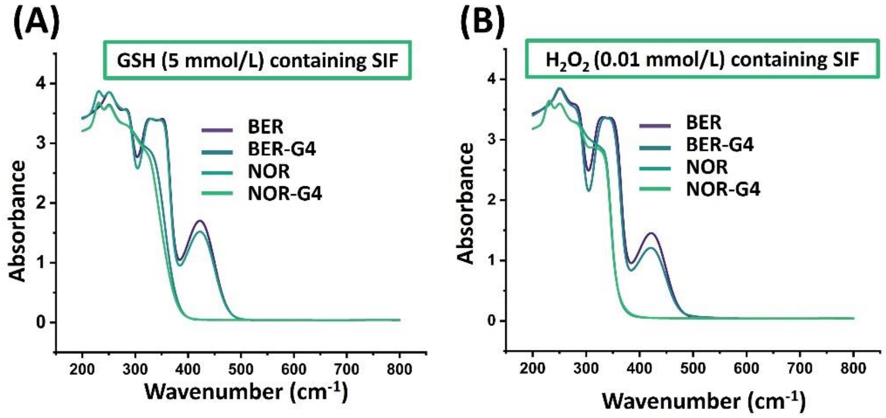

Figure S30 Stability of loaded BER/NOR during release. UV–Vis absorption spectra of bulk drug and drug released in SIF containing (A) GSH (5 mmol/L) or (B) $H_2O_2$0.01 mmol/L).

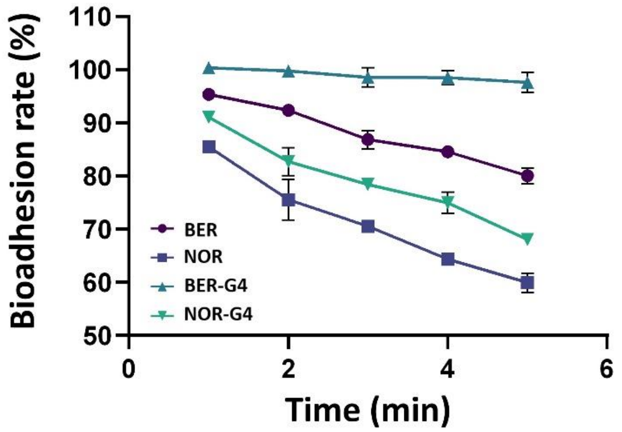

Figure S31 Bioadhesion capacityof the bulk drug and drug-loaded G4 on the intestinal mucosa under the elution of SIF. Data are presented as mean ± SD (*n* = 3).

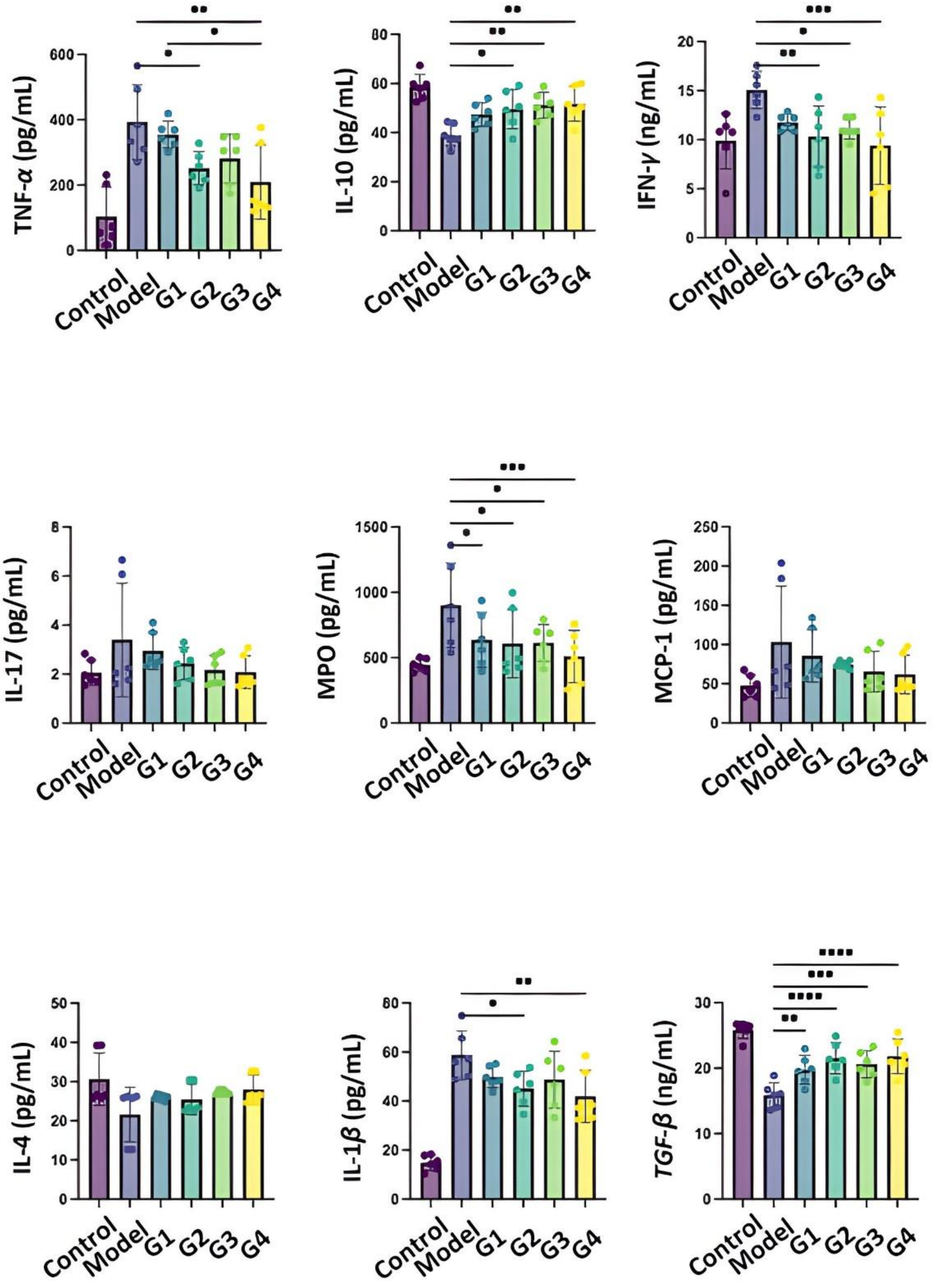

Figure S32 Pro- or anti-inflammatory cytokine levels for bacteria-infected mice before and after blank MSNs administration. Data are presented as mean ± SD (n = 6), *$P < 0.05$, **$P < 0.01$, ***$P < 0.001$, and ****$P < 0.0001$ *vs*. indicated.

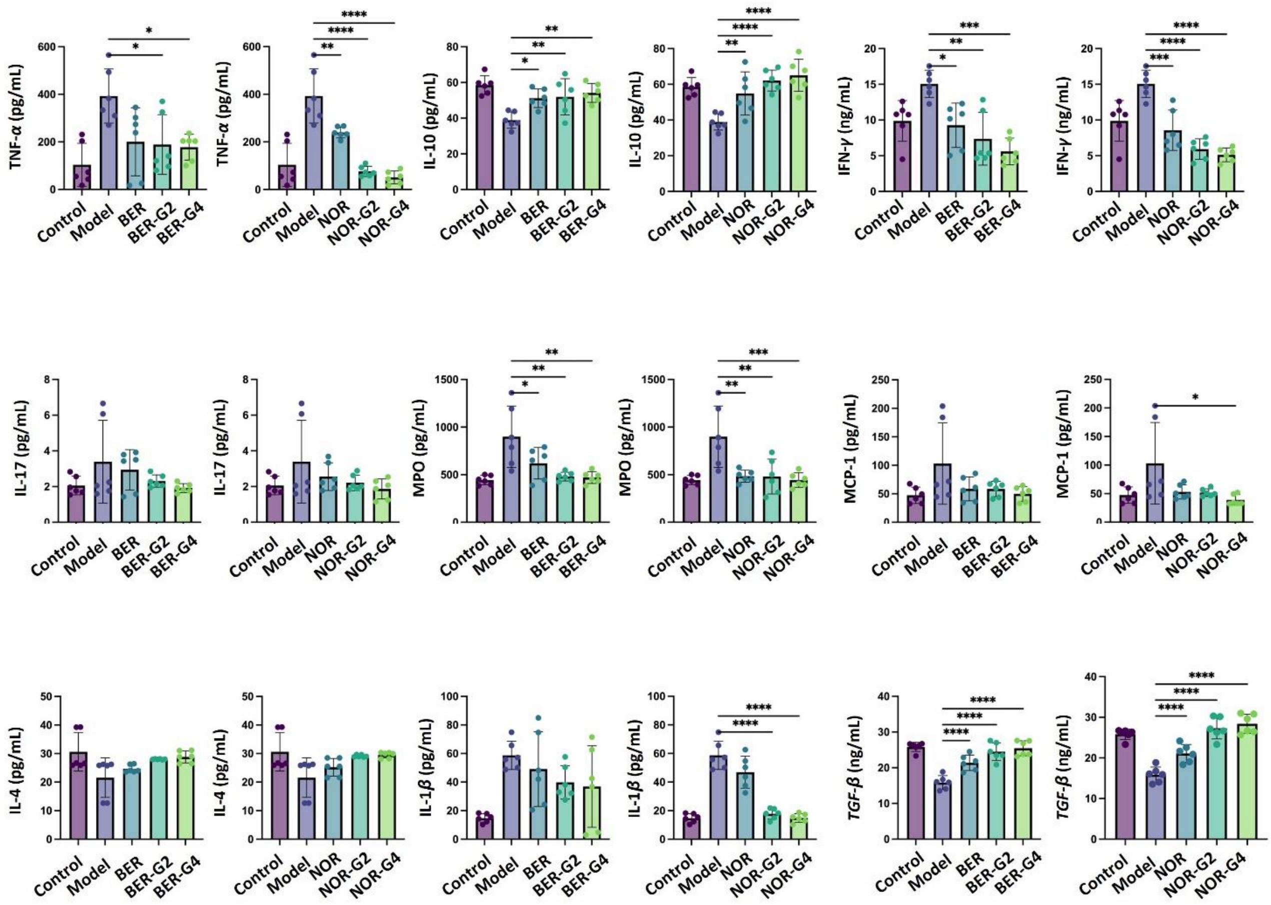

Figure S33 Pro- or anti-inflammatory cytokine levels for bacteria-infected mice before and after bulk drug, MSNs, or drug-loaded MSNs administration. Data are presented as mean ± SD, (n = 6), *P < 0.05, **P < 0.01, ***P < 0.001, and ****P < 0.0001 *vs*. indicated.

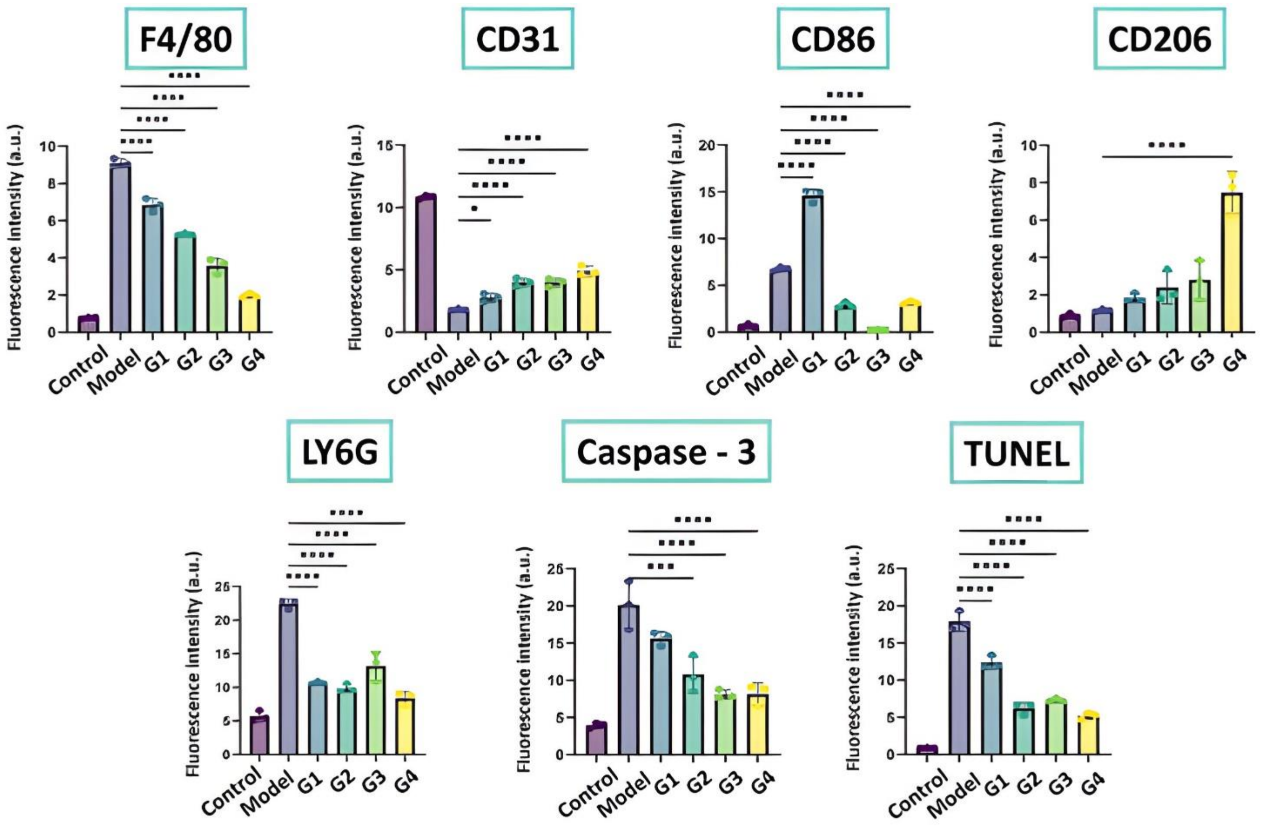

Figure S34 Semiquantitative fluorescence analysis of IF images from bacteria-infected mice before and after receiving blank MSNs. Data are presented as mean ± SD, (n = 3), *P < 0.05, ***P < 0.001, and ****P < 0.0001 *vs*. indicated. (λ_{ex}: 482 nm/λ_{em}: 536 nm for CD31, CD86, LY6G and TUNEL; λ_{ex}: 562 nm/λ_{em}: 624 nm for F4/80, CD206 and caspase-3).

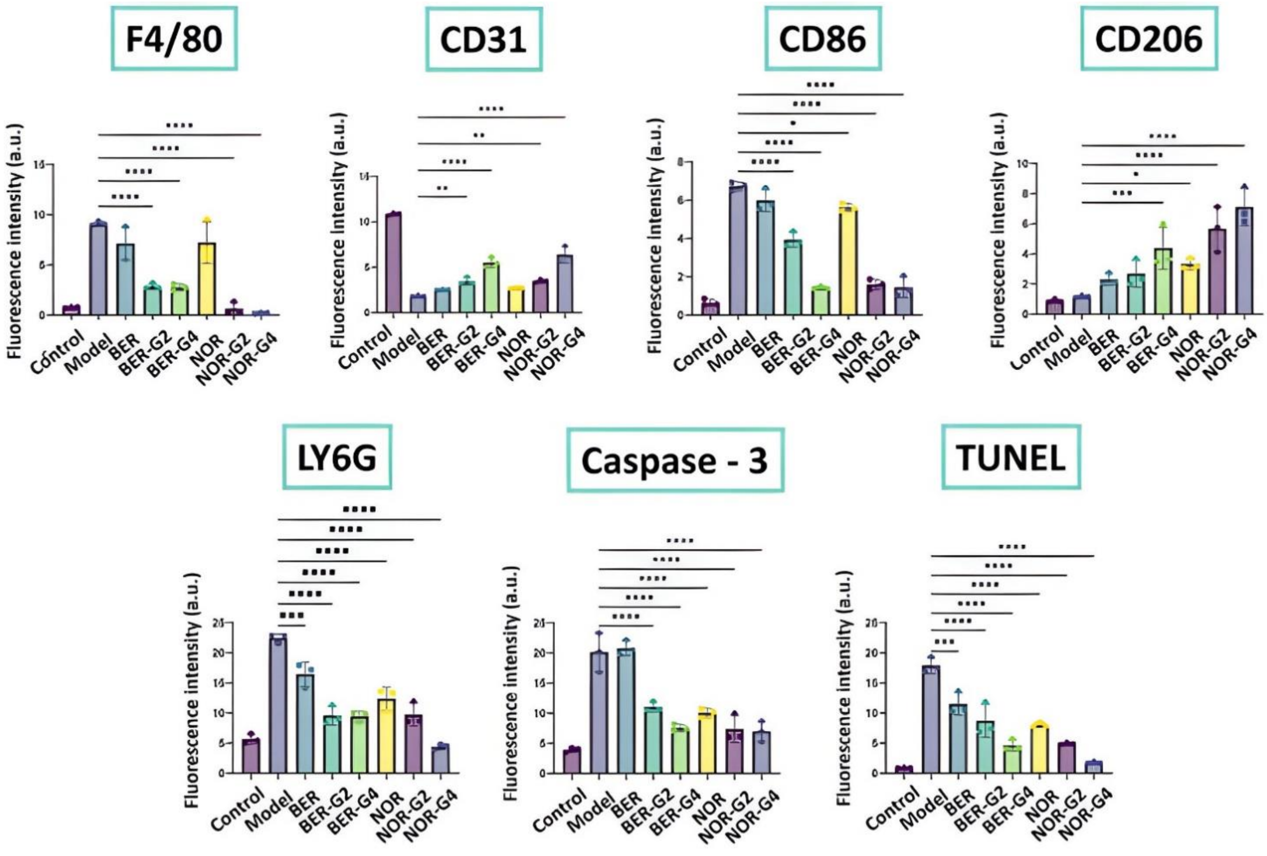

Figure S35 Semiquantitative fluorescence analysis of IF images from bacteria-infected mice before and after receiving bulk drug and drug-loaded MSNs. Data are presented as mean ± SD, (n = 3), *P < 0.05, **P < 0.01, ***P < 0.001, and ****P < 0.0001 *vs*. indicated. (λ_{ex}: 482 nm/λ_{em}: 536 nm for CD31, CD86, LY6G and TUNEL; λ_{ex}: 562 nm/λ_{em}: 624 nm for F4/80, CD206 and caspase-3).

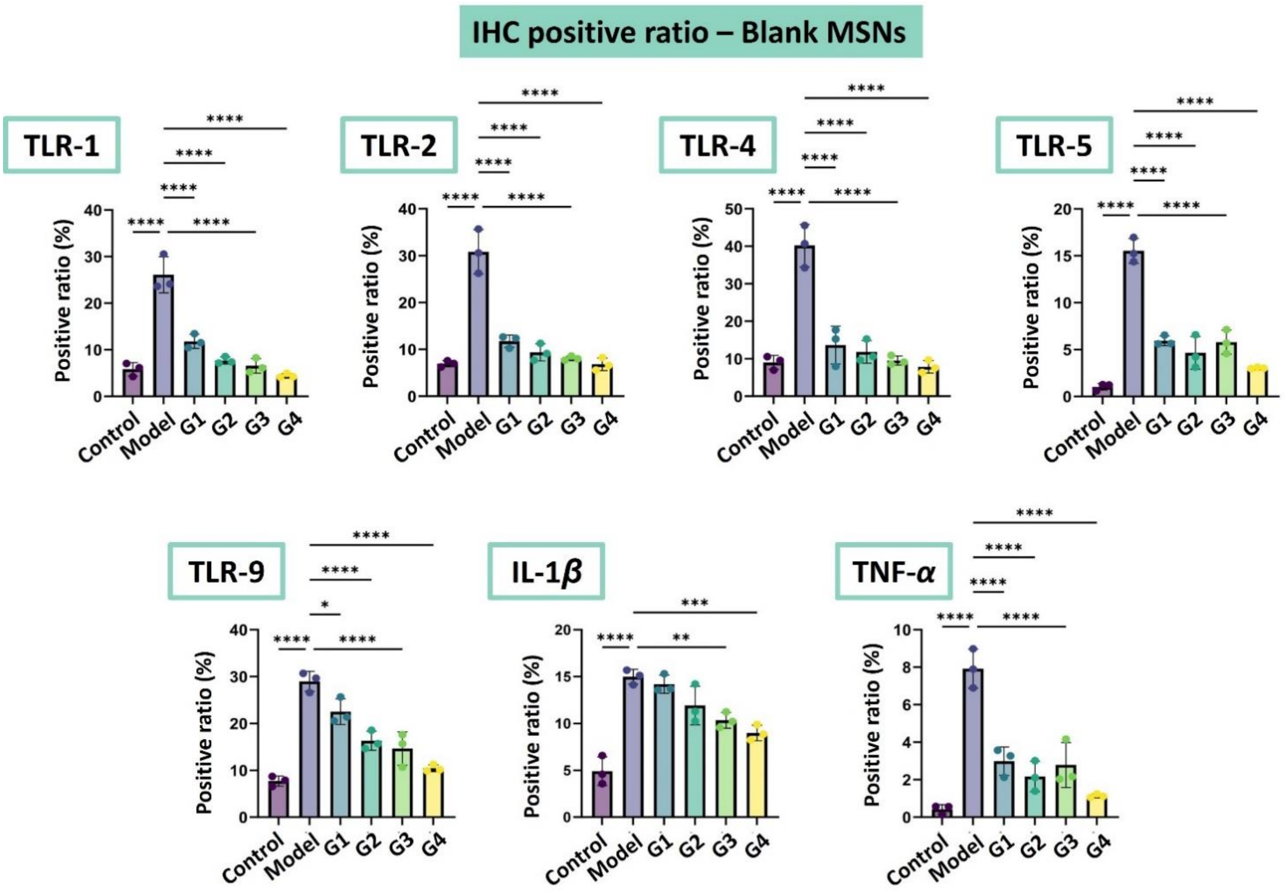

Figure S36 Semiquantitative analysis of IHC images from bacteria-infected mice before and after receiving blank MSNs. Data are presented as mean ± SD, (n = 3), *$P < 0.05$, **$P < 0.01$, ***$P < 0.001$, and ****$P < 0.0001$ *vs*. indicated.

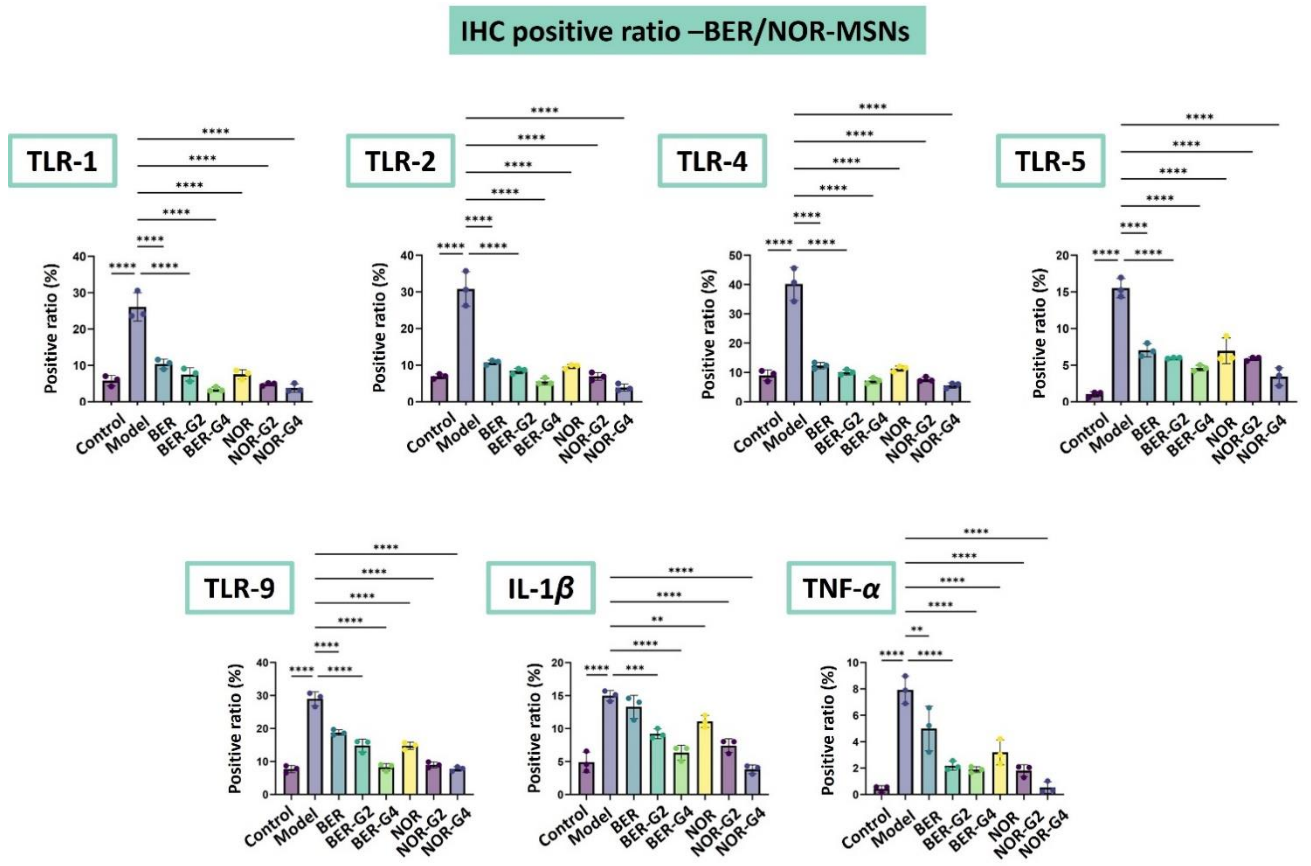

Figure S37 Semiquantitative analysis of IHC images from bacteria-infected mice before and after receiving bulk drug and drug-loaded MSNs. Data are presented as mean ± SD, (n = 3), $^{**}P < 0.01$, $^{***}P < 0.001$, and $^{****}P < 0.0001$ *vs*. indicated.

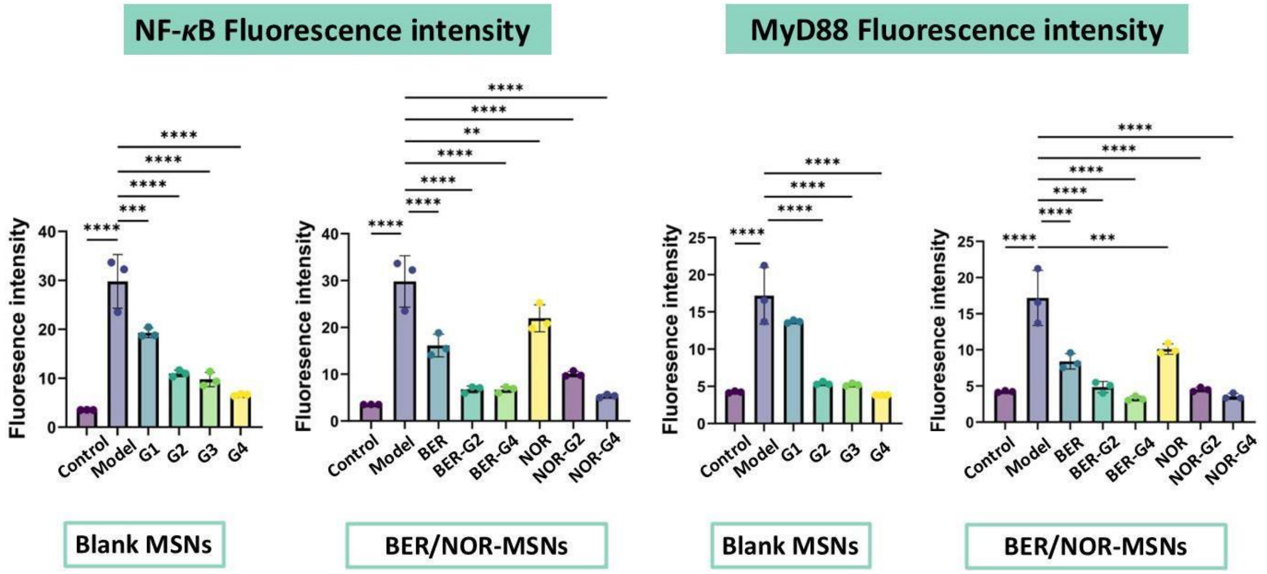

Figure S38 Semiquantitative fluorescence analysis of IF images (corresponding to NF-*κ*B and MyD88) from bacteria-infected mice before and after receiving blank MSNs. Data are presented as mean ± SD, ($n = 3$), $^{**}P < 0.01$, $^{***}P < 0.001$, and $^{****}P < 0.0001$ *vs*. indicated.

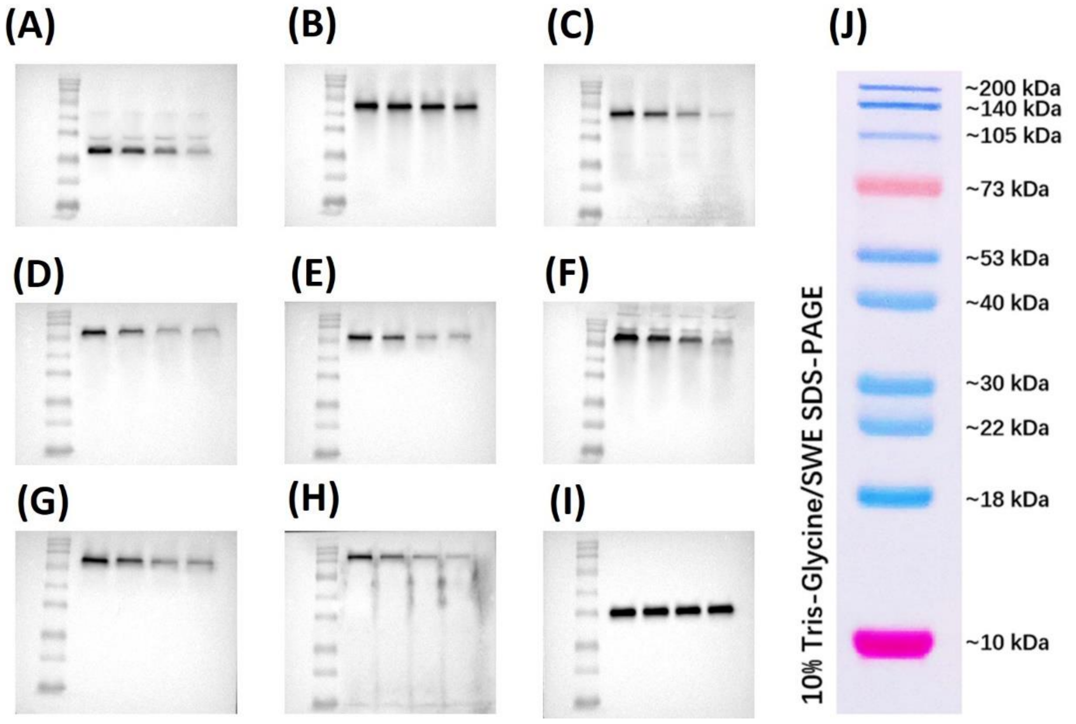

Figure S39 Raw data of western blotanalysis. (A) MyD88, (B) P65, (C) p-P65, (D) TLR-1, (E) TLR-2, (F) TLR-4, (G) TLR-5, (H) TLR-9, (I) *β*-Actin and (J) Molecular weight for corresponding protein marker. Groups were set as Model, G4, BER-G4, and NOR-G4 from left to right.

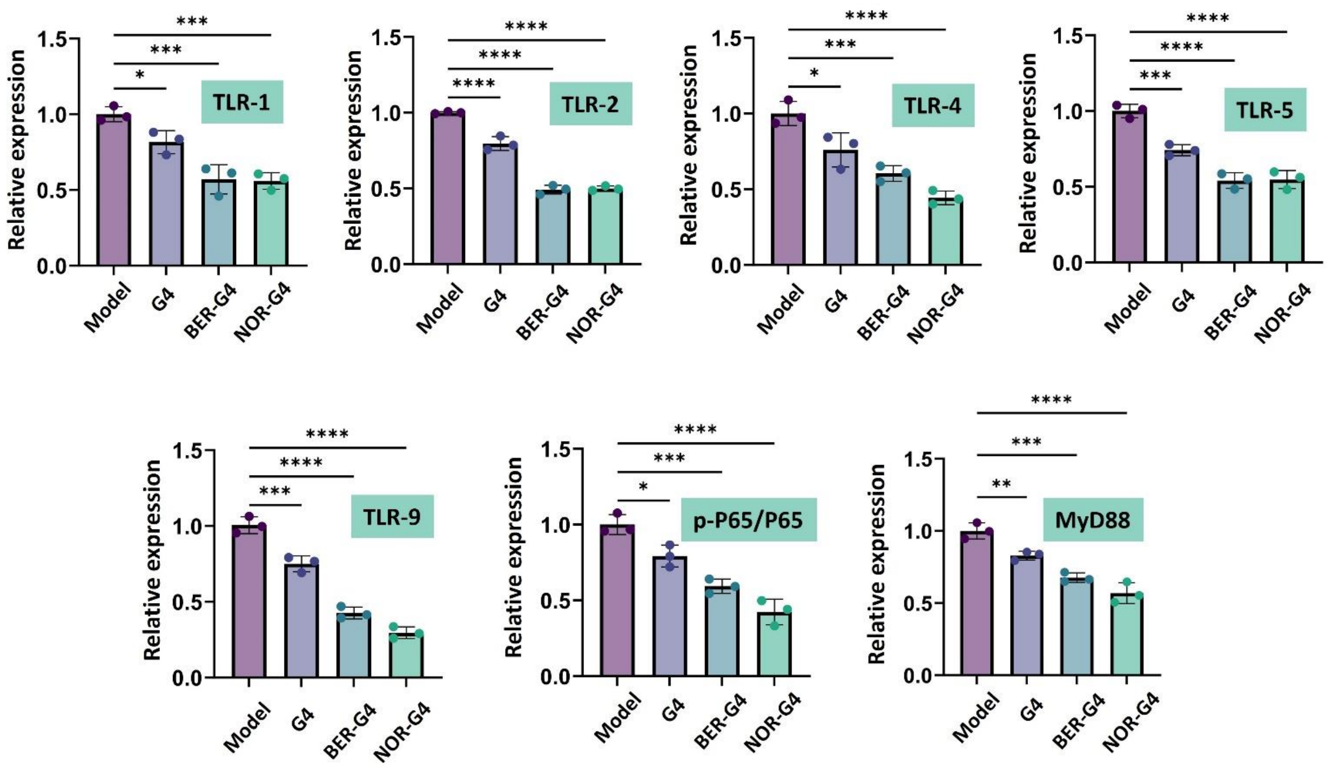

Figure S40 Quantitative analysis of the WB results. Data are presented as mean ± SD, (n = 3), *P < 0.05, **P < 0.01, ***P < 0.001, and ****P < 0.0001 *vs*. indicated.

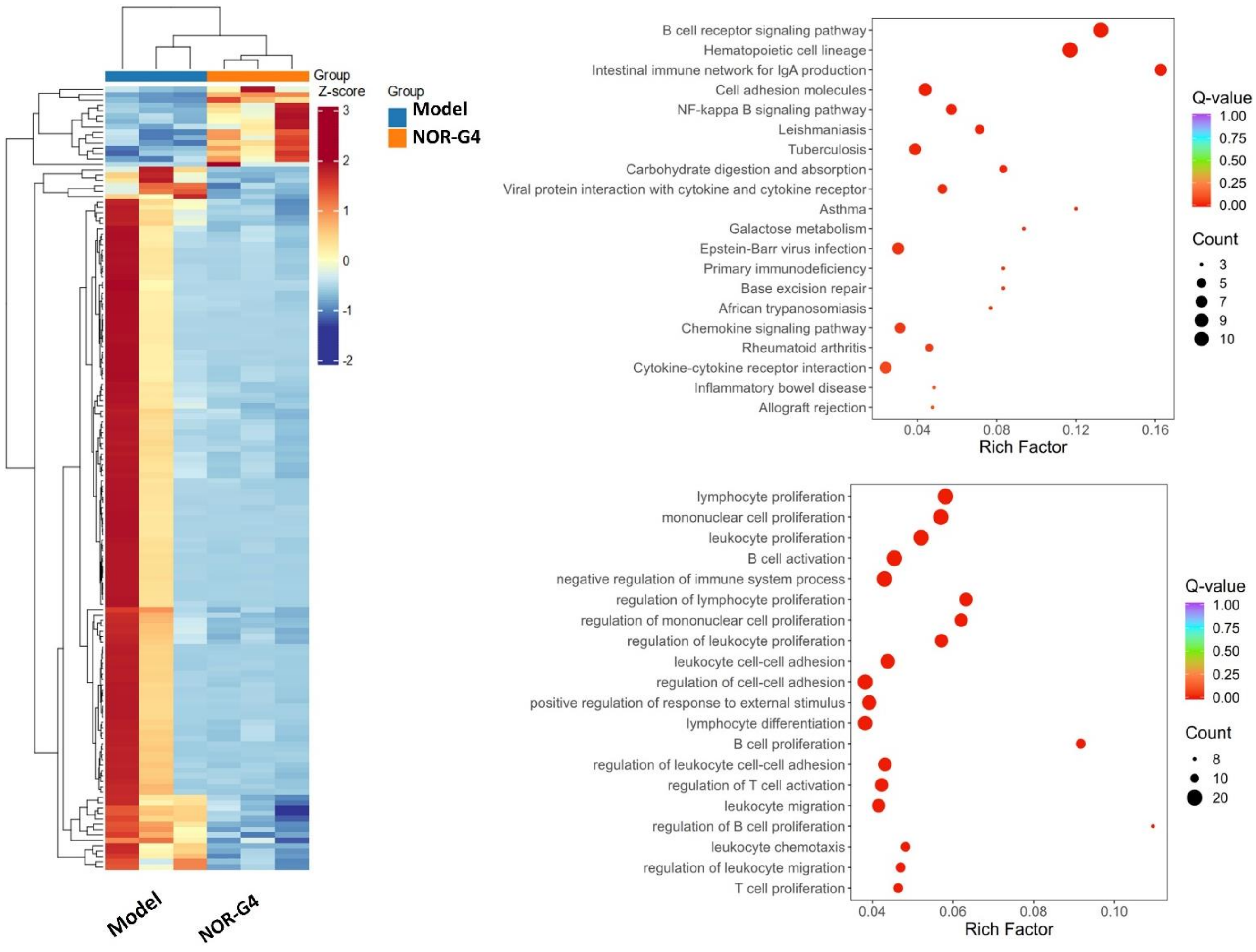

Figure S41 DEGs clustering heat map and KEGG scatter plot of intestine tissues of bacteria-infected mice before and after NOR-G4 treatment.

GO_level_1	GO	Description	DiffRatio
Biological process	GO:0046651	lymphocyte proliferation	20/133 15.04%
Biological process	GO:0032943	mononuclear cell proliferation	20/133 15.04%
Biological process	GO:0070661	leukocyte proliferation	20/133 15.04%
Biological process	GO:0042113	B cell activation	20/133 15.04%
Biological process	GO:0002683	negative regulation of immune system process	20/133 15.04%

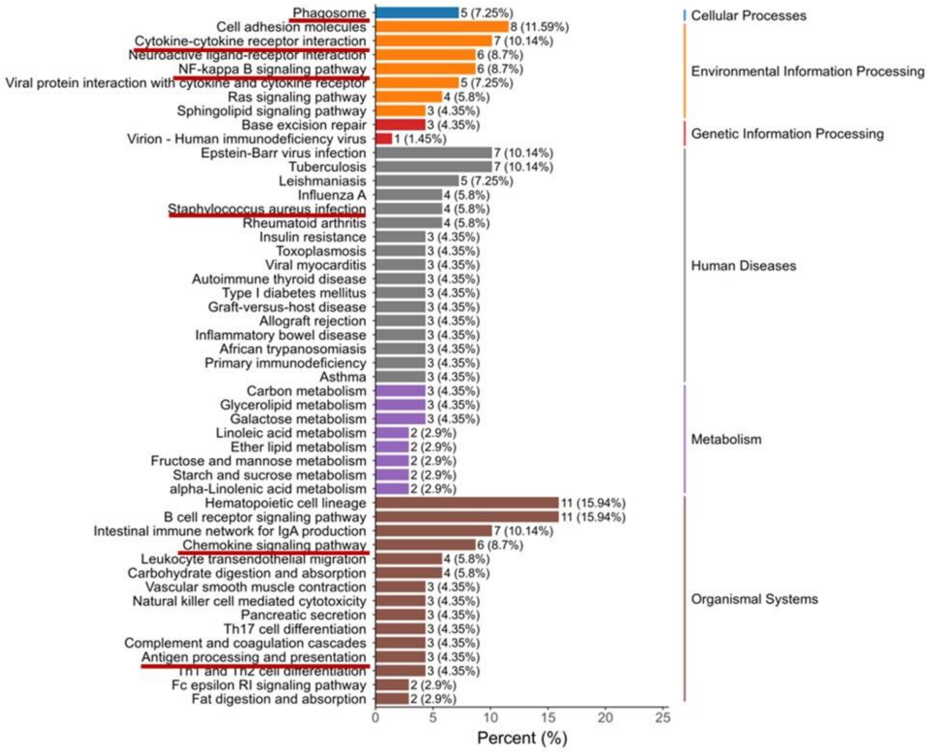

Figure S42 KEGG enrichment histogram and GO enrichment analysis of intestine tissues of bacteria-infected mice after NOR-G4 treatment.

Supporting tables

Table S1 Baseline characteristics of antibodies used in IF and IHC.

Antibodies	Company	Catalog No.	Clone Name	Lot No.	Dilution fold	Application
Anti-TLR1	Servicebio	GB11886	N/A	AC241016013	1:500	IHC
Anti-TLR2	Servicebio	GB113378	N/A	AC241016107	1:500	IHC
Anti-TLR4	proteintech	66350	3G9A4	N/A	1:600	IHC
Anti-TLR5	huabio	ET1703-30	JM10-88	H650064017	1:100	IHC
Anti-TLR9	abcam	ab134368	26C593.2	N/A	1:600	IHC
Anti-TNF-α	abcam	ab307164	RM1005	N/A	1:500	IHC
Anti-IL-1β	abcam	ab283818	RM1009	N/A	1:500	IHC
Anti-LY6G	abcam	ab238132	EPR22909-135	1073751-11	1:4000	IF
Anti-F480	CST	70076	D2S9R	9	1:600	IF
Anti-CD86	CST	19589	E5W6H	N/A	1:400	IF
Anti-CD206	CST	24595	E6T5J	N/A	1:1000	IF
Anti-caspase3	abcam	ab184787	EPR18297	N/A	1:3000	IF
Anti-CD31	abcam	ab281583	RM1006	N/A	1:1200	IF
Anti-MyD88	proteintech	67969	2E10D3	N/A	1:200	IF
Anti-NF-κB	abcam	ab32360	E381	N/A	1:750	IF
